# Within-individual variation of spirometry measurements in primary care: a retrospective cohort study

**DOI:** 10.1136/bmjresp-2025-003853

**Published:** 2026-05-17

**Authors:** Alex Gough, Tom Marshall, Alice M Turner, Alice Sitch

**Affiliations:** 1 Department of Applied Health Sciences, University of Birmingham, Birmingham, England, UK; 2 Birmingham Biomedical Research Centre, National Institute for Health and Care Research, London, England, UK; 3 Birmingham Biomedical Research Centre, National Institute for Health and Care Research, Birmingham, England, UK

**Keywords:** Respiratory Function Test, COPD epidemiology, Asthma Epidemiology

## Abstract

**Background:**

Forced expiratory volume in 1 s (FEV_1_) and forced vital capacity (FVC) are two of the most common measurements in spirometry, which are used for the diagnosis and monitoring of respiratory disease. Within-individual variation in measured FEV_1_ and FVC may affect their clinical utility but estimates of this are based on limited and often poor-quality data.

**Methods:**

A retrospective cohort study was performed. Data on FEV_1_ and FVC results and sociodemographic, lifestyle and comorbidity covariates were extracted from the IQVIA Medical Research Database. A minimum of four measurements in the same individual within a 6-month time period from the first measurement was the only inclusion criterion. Within-individual measured variation was calculated as a coefficient of variation (CV) using a linear regression random effects model using within-subject variance to estimate CV for the whole population and various subgroups.

**Results:**

4412 participants for FEV_1_ and 3567 participants for FVC were included in the main study. The mean number of measurements per individual in the 6-month period was 4.8 (SD 2.3) for FEV_1_ and 5.1 (SD 3.5) for FVC. The overall CV for FEV_1_ was 22.4% (95% CI 22.1% to 22.8%) and for FVC was 15.2% (95% CI 15.0% to 15.5%). This is much higher than seen in a previous systematic review of spirometry variation. CV increased as mean patient FEV_1_ and FVC decreased.

**Conclusions:**

Estimated within-individual variation in this analysis of real-world data is much higher than previously reported. Variation increases with more severe disease status. This has important implications for diagnosis, monitoring and clinical decision-making.

**WHAT IS ALREADY KNOWN ON THIS TOPIC**

- Previous studies have shown important evidence of within-individual variation in spirometry measures, but have tended to be based on small numbers and/or healthy populations. There are little published data on within-individual variation of forced expiratory volExpiratory Volume in 1 s (FEV_1_) and forced vital cForced Vital Capacity (FVC) in real-world primary care settings.

**WHAT THIS STUDY ADDS**

- This is the first large study that describes within-individual variation of FEV_1_ and FVC in a primary care setting, and it shows that variation is higher than previously reported. Further, it shows that misclassification of respiratory disease status based on FEV_1_ and FVC measurements due to within-individual variation may be more common than previously thought.

**HOW THIS STUDY MIGHT AFFECT RESEARCH, PRACTICE OR POLICY**

- Within-individual variation in FEV_1_ and FVC needs to be taken into account when creating guidelines for diagnosis and treatment thresholds and when diagnosing and monitoring respiratory disease by spirometry in practice.

## Introduction

 Spirometry is recommended for the diagnosis and management of asthma and chronic obstructive pulmonary disease (COPD).^1^ In England, it is commonly delivered in primary care. Concerns have been expressed over the technical quality and reliability of spirometry in this setting, although the real-world usefulness of the test is still considered to be high.^2^ Forced expiratory volume in 1 s (FEV_1_) and forced vital capacity (FVC) are two of the most common measurands assessed by spirometry. These are usually reported in absolute values of volume (L or mL) or in relative values (percentage of predicted value derived from reference tables that take into account height, age, sex and ethnicity).^3 4^ The ratio of FEV_1_/FVC is used to help diagnose airflow obstruction, with a threshold <0.7 traditionally cited as normal.^5^ Individual variation may differ between different subgroups of patients for example with age, health status, sex or ethnicity.

Current National Institute for Health and Care Excellence (NICE) guidelines suggest the presence of mild airflow obstruction is indicated by an FEV_1_ value of between 50% and 79% of the predicted value, with less than 30% indicating severe airflow obstruction (see online supplemental table S5).^6^

These guidelines acknowledge that spirometry testing, like all biological measurements, varies within an individual over time. This within-individual variation is broadly composed of three parts. Pre-analytical variation is variation due to varying factors prior to a measurement. For spirometry, this may include factors such as recent exercise, smoking or consumption of alcohol or a large meal.^7^ Analytical variation is the variation introduced by imprecision in the measuring process. In the case of spirometry, this may relate to the technical quality of the spirometry testing, the device, the operator and calibration. Biological variation is the within-individual variation, influenced partly by predictable factors such as season, time of day or monthly hormone cycles, but also to a large extent by chance. The sum of these three types of variation gives the total within-individual variation.^8^ The higher the within-individual variation, the lower the probability that a single measurement is reflective of the true mean. Coefficient of variation (CV) (SD/mean) is commonly used to describe variation and is often expressed as analytical variation (CV_A_) and within-individual biological variation (CV_I_) components. In this study, it is not possible to separate analytical from biological variation, so within-individual CV is referenced as CV_T_, the T referring to total within-individual variation, that is the sum of biological and analytical variation.

It is important to be aware of the within-individual variation of spirometry testing to quantify the probability of errors in diagnosis and management of respiratory illness. However, a recent systematic review of within-individual variation of spirometry that formed part of the doctoral thesis of the lead author found that most studies published on this subject had small numbers of participants and/or small numbers of repeat measurements.^9^ Within-session variation of spirometry is high, and American Thoracic Society guidelines recommend repeated tests are performed in order to achieve three acceptable measurements of FEV_1_ and FVC, with repeatability recommendations based on the difference between the largest and next largest measurements.^10^ However, less guidance is available about how often tests should be repeated on different days to overcome the problem of longer-term within-individual variation.

Spirometry measures can decline over time with age and disease progression. For example, patients with COPD can show a mean decline in FEV_1_ of 33–69 mL/year with some patients showing as rapid a decline as 200 mL/year.^11^ Variation over a long time period, therefore, includes both short-term within-individual variation and a long-term decline. Restricting analyses to measurements performed within a 6-month period therefore will provide an estimate of short-term variation.

## Aims

To use real-world data (ie, data from clinical sources rather than experimental studies) from the IQVIA Medical Research Database (IMRD) to describe the within-individual variation of FEV_1_, FVC and FEV_1_/FVC in a large cohort of patients and to describe any covariates that have an effect on the variation. To describe the probability of within-individual variation in FEV_1_ and FVC resulting in clinically important changes in FEV_1_ and FVC in order to inform clinical decision-making with regard to observed FEV_1_ and FVC changes in a patient.

## Methods

A population-based retrospective cohort study was undertaken using data obtained from the IMRD. IMRD incorporates data from The Health Information Network (THIN), a Cegedim Database. The reference made to THIN is intended to be descriptive of the data asset licensed by IQVIA. IMRD is a pseudoanonymised database of the electronic healthcare records of patients registered with general practices in the UK which use compatible practice management systems. Data collection for the database commenced in 2003.

Details of the database, setting, data extraction and methods used in this study have been published elsewhere,^12^ except as described below.

The study was reported according to the STrengthening the Reporting of Observational studies in Epidemiology statement for observational studies.

### Setting

Data were obtained from UK general practices registered with THIN/IMRD from database inception to 9 September 2021. Data extraction from the database was performed using the Dexter software^13^ and was completed on 9 September 2021. The date of the latest entry was 11 January 2021. The authors had no access to data that could identify individual participants.

### Ethical approval

IQVIA Medical Research data have been approved by the NHS Health Research Authority (NHS Research Ethics Committee ref 18/LO/0441) for the purpose of medical and public health research and to supply data for researchers for scientifically approved studies. Patients who have opted out of THIN or the IQVIA Medical Research Extraction Scheme will no longer be included in the data extracts from that point forward. The use of the data for this study was approved by the IQVIA Scientific Review Committee on 19 November 2020 with the reference 20SRC068.

### Patient and public involvement

There was no patient/public participation in this study.

### Participants

All participants in the database were eligible for inclusion. The primary analysis used all measurements within a 6-month period, for which there were at least four measurements on different days in order to estimate short-term variation rather than include variation due to the long-term decline with age or disease progression. Participants were excluded if they did not have at least four recorded measurements of FEV_1_ or FVC recorded in a 6-month period.

**Figure 1:**
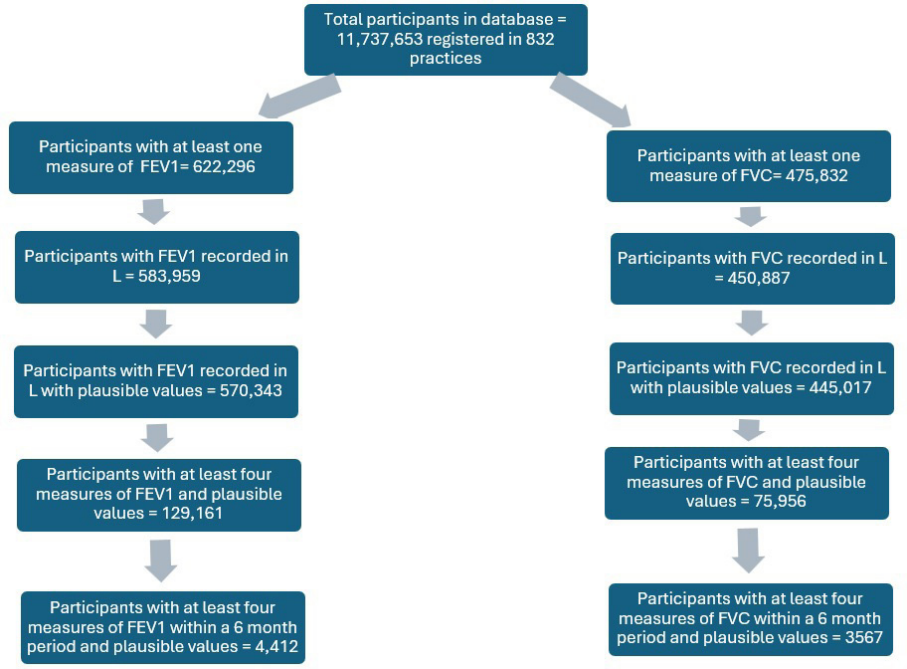
Participant selection algorithm. FEV1, forced expiratory volume in 1 s; FVC, forced vital capacity.

The study size included the entire database where participants had four or more measurements of FEV_1_ and FVC within 6 months of the first measurement (see figure 1).

### Variable choice

The coprimary outcomes were within-individual variation of FEV_1_ and FVC as demonstrated by CV_T_. Other variables included in analyses are shown in online supplemental table S6. Variables were selected as those hypothesised most likely to have an effect on variation based on internal expert review or that enabled calculation of variation (eg, date of measurement). The date of each FEV_1_ or FVC test and the date of diagnosis where applicable were also extracted. Date of covariables such as body mass index (BMI) did not necessarily match the date of the measurement. It was not possible to distinguish between prebronchodilator and postbronchodilator measurements in the database. However, NICE guidance does not recommend reversibility testing for routine diagnosis and monitoring, therefore it is assumed that most of the measurements are prebronchodilator.^6^ Note, though, that without specific acute bronchodilator administration, many patients (particularly with COPD) would be taking regular long-acting bronchodilators which might have been taken as per normal.

### Data cleaning

Most of the spirometry measurements were reported in L so those expressed as a per cent predicted were excluded from the main analysis since the units are not interchangeable with regards to calculation of CV (but values recorded as per cent predicted were used for a sensitivity analysis). Height data were not available so per cent predicted could not be back-calculated. Values of FEV_1_ or FVC of 0 L or less, or >10 L were excluded as being implausible based on published data, for example,^14^ an analysis of the IMRD data.

### Statistical methods

Statistical calculations were performed using Stata V.17.0. CV_T_ is expressed as percentage. FEV_1_/FVC was calculated by dividing the results for FEV_1_ by those for FVC on days where participants had had both these measurements performed. Within-individual variance was estimated using a random effects model (‘mixed’ command), which uses a random intercept for grouping variables, with a restricted maximum likelihood. A normal distribution was assumed based on visual examination of the data, so the data were not log transformed. A CV was then calculated from the SD and mean using the formula CV=SD/mean × 100%. Means and 95% CIs were estimated from the model in Stata. Heteroscedasticity was evaluated by visual inspection of standardised residuals versus fitted values. Reference change values (RCV) were calculated with the formula RCV=z*√2*CV. The Repeatability Coefficient (RC) was calculated as RC=z*√2*SD. For both these calculations, z=1.96 for 95% confidence. Sample statistical code is included in online supplemental material Appendix 3. Univariable subgroup analyses were performed by age, sex, BMI, Townsend neighbourhood deprivation score quintiles,^15^ smoking and alcohol status, geographical location, mean and median FEV_1_ or FVC result and the presence of COPD, asthma and other comorbidities. Univariable subgroup analysis was also performed for ethnicity, but is not reported due to high levels of missingness in the data and low numbers of some ethnicities. Based on the variation calculated in the univariable analysis and the likely importance of factors in respiratory disease (eg, smoking status), several subgroups were selected to be combined into a multiple subgroup analysis to describe how within-individual variation of spirometry measurements differs between subgroups of individuals.

Various sensitivity analyses were performed to assess bias due to factors which might affect the robustness of the results such as our statistical methods utilising linear regression, number of repeat measurements and unit of measurement. An analysis was performed by using all the measurements in the database regardless of timing, that is including those measurements that were more than 6 months after the first measurement.

## Results

### Participants

The database included 11 737 653 participants registered in 832 practices. 622 296 participants had at least one FEV_1_ and 475 832 had at least one FVC. Figure 1 shows the flowchart of participant selection. After exclusion of measures not recorded in L and implausible values, 4412 participants had at least four measures of FEV_1_ within a 6-month period and 3567 participants had at least four measures of FVC within a 6-month period. The mean number of measurements per individual in the 6-month period was 4.8 (SD 2.3) for FEV_1_ and 5.1 (SD 3.5) for FVC. The mean interval between measurements was 28.0 days (SD 15.9 days) for FEV_1_ and 25.6 days (SD 16.3) for FVC.

Age and sex were complete throughout the data set. Participants with missing FEV_1_ and FVC results were not eligible. Where other comorbidities were not reported, they were assumed to be absent. Participants with missing data for other characteristics, such as BMI or deprivation score, were included in the main analysis but excluded from the subgroup analysis where these variables were used.

### Descriptive data and outcome data

CV_T_ for FEV_1_ was 22.4% (95% CI 22.1% to 22.8%) with a mean FEV_1_ of 2.03 L. The RCV was 0.62. CV_T_ for FVC was 15.2% (95% CI 15.0% to 15.5%) with a mean FVC of 3.03 L. The RCV was 0.42. For FEV1/FVC CV_T_ was 17.9% (95% CI 17.5% to 18.5%). Tables1^3^ show the participant characteristics and main univariable group analyses for FEV_1_, FVC and FEV_1_/FVC. Further univariable group analyses are found in online supplemental tables S1a and S1b and online supplemental figures S2 to S17. For FEV_1_, 4412 participants were included in the study, with a median age at first measurement of 72 (IQR 61–81), 54.5% being male. For FVC, 3567 participants were included in the study, with a median age at first measurement of 69 (IQR 57–79)*,* 53.9% being male.

**Table 1:** Sociodemographic data and main univariable subgroup analyses for FEV1

		N	N(%)	Mean	CV (%)	95% CI
	All	4412	100	2.03	22.4	22.1 to 22.8
Sex	Male	2404	54.5	2.29	22.9	22.4 to 23.4
	Female	2008	45.5	1.71	20.6	20.2 to 21.1
Age (years)	>10–20	39	0.9	2.14	15.3	12.7 to 17.8
	>20–30	89	2	2.77	15.9	14.2 to 17.6
	>30–40	146	3.3	2.9	13.1	12.1 to 14.1
	>40–50	255	5.8	2.85	19	18.0 to 20.1
	>50–60	537	12.2	2.49	21.5	20.6 to 22.3
	>60–70	910	20.7	2.16	22.6	21.9 to 23.3
	>70–80	1207	27.5	1.88	23.3	22.6 to 23.9
	>80–90	872	19.9	1.61	26	25.1 to 26.9
	90–100	331	7.5	1.39	24.7	23.4 to 26.1
BMI (kg/m^2^)	<18.5	99	1.4	1.88	27	23.8 to 30.2
	18.5–<25	1354	19.4	1.97	23.8	23.1 to 24.5
	25–<30	1285	18.4	2.08	22.2	21.6 to 22.8
	30–<35	474	6.8	2.01	21.2	20.3 to 22.2
	>35	273	3.9	2	20.8	19.6 to 22.1
	Missing	3486	50	2.02	22.7	22.3 to 23.1
Smoker status	Current	1651	37.4	1.97	25.3	24.6 to 25.9
	Ex	1031	23.4	1.95	23.8	23.0 to 24.6
	Never	1164	26.4	2.13	18.5	17.9 to 19.1
	Missing	566	12.8	–	–	–
Respiratory status	Normal	517	13.5	2.47	15.1	14.5 to 15.8
	Asthma	1805	47.1	2.05	21.6	21.0 to 22.1
	COPD	1514	39.5	1.72	29.1	28.4 to 29.9
	COPD and asthma	606	–	1.65	29.2	28.0 to 30.5

Mean FEV_1_ is in L. N=4412 except: for age, N=4386 as <10 years and >100 years of age are excluded; for respiratory status, N=3836*. *

BMI, body mass index; COPD, chronic obstructive pulmonary disease; CV, coefficient of variation; FEV1, forced expiratory volume in 1 s.

**Table 2:** Sociodemographic data and main univariable subgroup analyses for FVC

		N	N (%)	Mean	CV (%)	95% CI
	All	3567	100	3.03	15.2	15.0 to 15.5
Sex	Male	1921	53.9	3.51	14.9	14.6 to 15.2
	Female	1646	46.1	2.47	15.2	14.9 to 15.6
Age (years)	>10–20	43	1.2	2.55	12.7	10.8 to 14.7
	>20–30	86	2.4	3.54	11.5	10.3 to 12.7
	>30–40	146	4.1	3.85	11.8	10.9 to 12.7
	>40–50	247	7.0	3.86	12.6	11.9 to 13.3
	>50–60	461	13.0	3.55	14.2	13.6 to 14.8
	>60–70	800	22.5	3.19	15.3	14.8 to 15.8
	>70–80	924	26.0	2.89	15.9	15.4 to 16.3
	>80–90	630	17.8	2.44	17.6	16.9 to 18.2
	90–100	211	5.9	2.13	17.5	16.4 to 18.7
BMI (kg/m^2^)	<18.5	72	2.0	2.78	14.8	12.9 to 16.6
	18.5–<25	1101	30.9	3.02	15.6	15.1 to 16.0
	25–<30	976	27.4	3.07	15	14.6 to 15.5
	30–<35	428	12	2.96	14.9	14.2 to 15.6
	>35	208	5.8	2.78	17.8	16.7 to 18.9
	Missing	782	21.9	3.12	14.5	14.0 to 15.1
Smoker status	Current	1289	36.1	3.03	15.8	15.4 to 16.2
	Ex	808	22.7	2.94	15.6	15.1 to 16.1
	Never	1003	28.1	3.04	14.6	14.1 to 15.0
	Missing	493	13.8	–	–	–
Respiratory status	Normal	489	16.3	3.26	13.6	13.0 to 14.2
	Asthma	1422	47.4	3.08	14.9	14.5 to 15.3
	COPD	1087	36.3	2.8	17.5	16.9 to 18.0
	COPD and asthma	403	–	2.7	18.4	17.5 to 19.3

Mean FVC is in L. N=3567 except: for age, N=3548 as <10 years and >100 years of age are excluded; for respiratory status, N=2998*.*

BMI, body mass index; COPD, chronic obstructive pulmonary disease; CV, coefficient of variation; FVC, forced vital capacity.

**Table 3:** Sociodemographic data and main univariable subgroup analyses for FEV1/FV

		N	N (%)	Mean	CV (%)	95% CI
	All	1166	100	0.68	17.9	17.5 to 18.4
Sex	Male	670	57.5	0.66	16.2	15.6 to 16.7
	Female	496	42.5	0.70	19.8	19.0 to 20.5
Age (years)	>20–30	22	1.9	0.80	11.7	08.3 to 12.0
	>30–40	57	4.9	0.78	10.2	28.4 to 35.5
	>40–50	65	5.6	0.73	31.9	10.6 to 12.9
	>50–60	113	9.8	0.75	11.7	11.2 to 13.1
	>60–70	201	17.4	0.72	12.1	14.4 to 16.2
	>70–80	311	27.0	0.67	15.3	16.3 to 17.9
	>80–90	280	24.3	0.66	17.1	18.8 to 20.9
	90–100	104	9.0	0.67	19.8	17.0 to 20.1
BMI (kg/m^2^)	<18.5	31	2.7	0.66	54.4	42.6 to 62.2
	18.5–<25	345	29.6	0.65	18.9	18.0 to 19.8
	25–<30	338	29.0	0.69	13.6	13.0 to 14.3
	30–<35	124	10.6	0.71	15.7	14.5 to 16.8
	>35	67	5.7	0.76	13	11.7 to 14.3
	Missing	261	22.4	0.67	15.5	14.7 to 16.3
Smoker status	Current	386	33.1	0.65	20.1	19.2 to 21.0
	Ex	287	24.6	0.65	16.5	15.7 to 17.3
	Never	329	28.2	0.73	17.5	16.7 to 18.3
	Missing	164	14.1	–	–	–
Respiratory status	Normal	118	11.2	0.78	8.2	7.6 to 8.8
	Asthma	556	53.0	0.67	18.2	17.5 to 18.8
	COPD	376	35.8	0.61	22.9	21.8 to 23.9
	COPD and asthma	162	–	0.58	18.6	17.2 to 19.9

Mean FEV1/FVC is a unitless ratio.N= 1166 except: for age, N=1153 as <20 years and >100 years of age are excluded; for respiratory status, N=1050.

BMI, body mass index; COPD, chronic obstructive pulmunary disease; CV, coefficient of variation; FEV1, forced expiratory volume in 1 s; FVC, forced vital capacity.

For FEV_1_, data were recorded as ‘missing’ in Townsend Deprivation Score for 454/4412 (10.3%) participants. For FVC, data were recorded as ‘missing’ in Townsend Deprivation Score for 373/3567 (10.5%) participants.

#### Subgroup analyses

**Figure 2:**
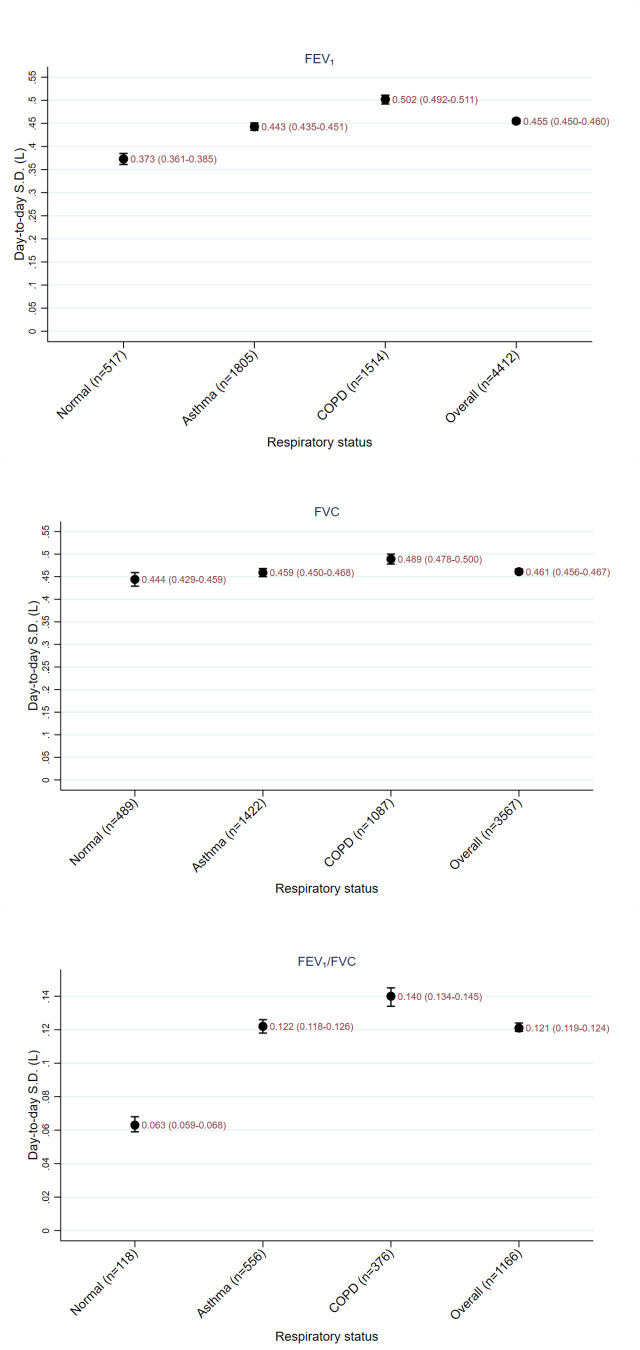
Graph of CV_T_ of FEV_1_, FVC and FEV_1_/FVC by respiratory status with error bars showing 95% CIs. COPD, chronic obstructive pulmonary disease; CV_T_, within-individual coefficient of variation; FEV1, forced expiratory volume in 1 s; FVC, forced vital capacity.

Table 4 and figures2  3 show the variation of FEV_1_, FVC and FEV_1_/FVC according to respiratory disease status, as described by CV and SD. Online supplemental table S9 shows the RC by respiratory disease status and the likelihood of the true change being in the direction observed given at different CIs.

**Table 4:** Day-to-day variability in spirometry values Note that N for the individual groups is slightly less than overall N due to the exclusion of a small number of patients who had measurements before and after a change in disease status

	N	Day-to-day variability in FEV_1_ CV in % (95% CI)	Day-to-day variability in FEV_1_ SD in L (95% CI)
No respiratory disease	517	15.1 (14.5 to 15.8)	0.373 (0.361 to 0.385)
Asthma	1805	21.6 (21.0 to 22.1)	0.443 (0.435 to 0.451)
COPD	1514	29.1 (28.4 to 29.9)	0.502 (0.492 to 0.511)
Asthma and COPD	606	29.2 (28.0 to 30.5)	0.481 (0.466 to 0.496)
Overall	4412	22.4 (22.1 to 22.8)	0.455 (0.450 to 0.460)

		Day-to-day variability in FVC CV in % (95% CI)	Day-to-day variability in FVC SD in L (95% CI)
No respiratory disease	489	13.6 (13.0 to 14.2)	0.444 (0.429 to 0.459)
Asthma	1422	14.9 (14.5 to 15.3)	0.459 (0.450 to 0.468)
COPD	1087	17.5 (16.9 to 18.0)	0.489 (0.478 to 0.5000
Asthma and COPD	403	18.4 (17.5 to 19.3)	0.495 (0.477 to 0.513)
Overall	3567	15.2 (15.0 to 15.5)	0.461 (0.456 to 0.467)

		Day-to-day variability in FEV_1_/FVC CV in % (95% CI)	Day-to-day variability in FEV_1_/FVC SD (unitless ratio) (95% CI)
No respiratory disease	118	8.2 (7.6 to 8.8)	0.063 (0.059 to 0.068)
Asthma	556	18.2 (17.5 to 18.8)	0.122 (0.118 to 0.126)
COPD	376	22.9 (21.8 to 23.9)	0.140 (0.134 to 0.145)
Asthma and COPD	162	18.6 (17.2 to 19.9)	0.108 (0.101 to 0.114)
Overall	1166	17.9 (17.5 to 18.4)	0.121 (0.119 to 0.124)

COPD, chronic obstructive pulmunary disease; CV, coefficient of variation; FEV1, forced expiratory volume in 1 s; FVC, forced vital capacity.

**Figure 3:**
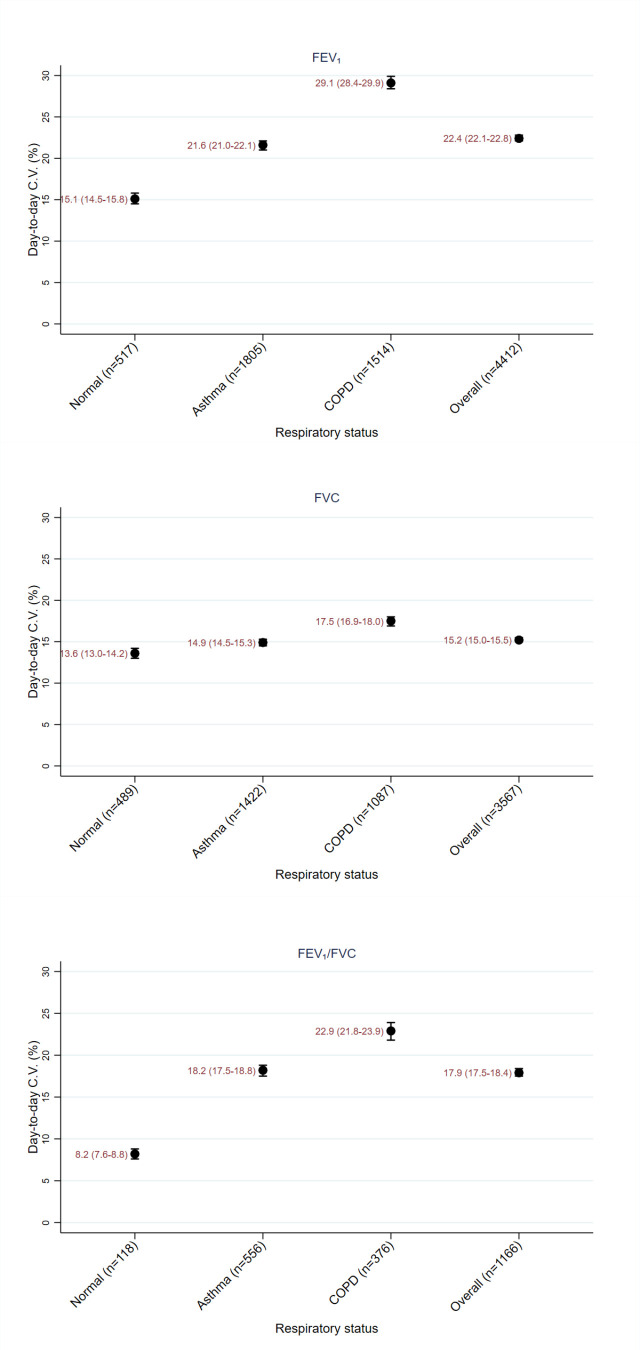
Graph of SD of FEV_1_, FVC and FEV_1/_FVC by respiratory status with error bars showing 95% CIs. COPD, chronic obstructive pulmonary disease; CV, coefficient of variation; FEV1, forced expiratory volume in 1 s; FVC, forced vital capacity.

Subgroup analysis of CV_T_ was also performed by sex, age, geographical region, Townsend Deprivation Score, BMI, presence of comorbidities, alcohol consumption and smoking status (see online supplemental information online supplemental figures S5 to S22).

For FEV_1_, higher CV_T_s were seen in the following subgroups: males compared with females; ex-drinkers and lifelong teetotallers compared with current drinkers; current smokers and ex-smokers compared with participants who have never smoked; participants with higher deprivation scores (although number of participants in some of these subgroups was low and the error bars were large). There was an increase in CV_T_ with increasing age. Participants with higher BMIs tended to have lower CV_T_s. For subgroups based on comorbidities, COPD and heart failure had the highest CV_T_s and no comorbidity or diabetes had the lowest CV_T_s. There was no obvious pattern in the differences of CV_T_ seen between patients from different regions.

Similar findings were seen for FVC except: there was little difference in CV_T_ between sexes; a less clear increase in CV_T_ with deprivation score; no obvious difference in CV_T_ with BMI.

Variation increased with decreasing mean and median FEV_1_ and FVC levels, that is variation increased with worsening disease status (online supplemental figures S1a, S1b, S32 and S33).

Online supplemental tables S2a and S2b show multiple subgroup analyses. These suggest that respiratory diagnosis has the largest effect on variation.

#### Sensitivity analyses

Sensitivity analyses performed for CV_T_ by year, quarter, number of measurements, implausible values, median FEV_1_ and FVC level, number of days between measurements, results coded as L v % and a crude estimate of CV_T_ using an arithmetic method rather than a linear regression model for calculation and using long-term measurements are reported in online supplemental table S3a and S3b and figures S18–S34.

For FEV_1_, there was little difference in CV_T_ when calculated by a simple arithmetic method compared with the linear regression model. CV_T_ was lower when calculated for results recorded in units of per cent predicted compared with L, but the number of participants for per cent predicted was lower (4412 for L and 515 for % predicted). There was no evidence of marked seasonality or a trend over years for the population CV_T_. Data stratified by patient median or mean were similar except for very low values. A smaller interval between measurements was associated with a higher CV_T_ and a slight increase in CV_T_ with a larger number of measurements, consistent with more measurements being performed when disease is less stable.

There was little difference in findings from the short-term results when a long-term analysis involving all recorded measurements was performed (online supplemental tables S4a and S4b and online supplemental figures S32–34). A smaller number of measurements over a long or short time period did not greatly affect the CV_T_ (online supplemental tables S7 and S8).

FVC CV_T_ had similar findings to the analyses for FEV_1_.

## Discussion

### Key results

This is one of the largest studies of between-day within-individual variation of FEV_1_ to date, with 4412 participants for FEV_1_ and 3567 for FVC. A previous systematic review found 104 studies reporting within-individual variation of FEV_1_ and/or FVC, but most had small numbers of participants and/or small numbers of repeat measurements.^9^

The previous largest study of between-day within-individual variation of FEV_1_ and FVC was performed in 1995 and consisted of only two measurements.^13^ The previous systematic review reported median CV_T_s of 2.8% for FEV_1_ and 2.9% for FVC for healthy populations; 6.8% for FEV_1_ and 6.8% for FVC for patients with COPD and 3.0% for FEV_1_ and 2.4% for FVC for patients with asthma.^9 16^

In the current study, the overall CV for FEV_1_ was 22.4% (95% CI 22.1% to 22.8%) with a mean of 2.03 L and for FVC was 15.2% (95% CI 15.0% to 15.5) with a mean of 3.03 L. This is much higher than seen in the systematic review. For participants with COPD, asthma or both conditions, the CV_T_s of FEV_1_ and FVC were also much higher than seen in the systematic review.

In this study, higher CV_T_ of spirometry measures was associated with having a higher deprivation score, being a current or ex-smoker, being older and having COPD or heart failure. Factors such as age, presence of COPD and smoking history are likely to affect the pathological variation in spirometry measures due to their effect on lung function and may also to some extent be interdependent. Participants with higher BMI tended to have lower variation. Previous studies are contradictory as to whether BMI has an effect on spirometry results, possibly related to whether populations were healthy or had COPD. However, one study showed that median FEV_1_ was higher in patients with higher BMI. Since in this study, a higher median FEV_1_ is associated with a lower variation, the lower variation in patients with higher BMI may relate purely to the difference in median FEV_1_.^17 18^ The median age for participants in this study was relatively high at 72 (for the FEV_1_ analysis), which might be associated with an increased pathological variability due to increased incidence and severity of COPD and other comorbid conditions in older patients.

CV_T_ was seen to increase as mean FEV_1_ or FVC decreased. This suggests that patients with more severe disease status had increased variation of spirometry values. This is an important finding, since patients with more severe disease may have less accurate results, making treatment decisions harder.

The multiple subgroup analyses (online supplemental tables S2a and S2b) show that disease status is an important factor associated with CV_T_, with patients with COPD having higher CV_T_ than healthy patients and patients with asthma being intermediate. This later finding is surprising since asthma is, by nature, a variable condition, while COPD is supposed to have a fixed airflow obstruction. This might be explained by the facts that: theoretically, all spirometry should be performed when the patient is in a stable condition, since variability in control might lead to no measure being made; COPD patients tend to be older and more likely to be smokers which could increase variability.

### Strengths and limitations

One strength of this study is the large size of the dataset, although the number of participants was greatly reduced by the decision to restrict analyses to short-term measurements. However, sensitivity analyses with longer-term measurements involving much higher numbers of participants gave similar results (see online supplemental tables S4a and S4b). It is also relevant that the data are real-world data taken from the clinical records of primary care practices in the UK and not measurements taken under ideal or experimental conditions. The Biological Variation Data Critical Appraisal Checklist recommends biological variation studies are performed on patients in steady state, with ‘preanalytical procedures described and standardised to minimise preanalytical variation’.^19^ This is important when using biological variation data to set analytical performance specifications, but is less relevant to clinical decision-making.

Inclusion of the entire dataset meant the only potential bias introduced into the results (that was not already present in the database) could come from data cleaning (which reduces errors introduced at data entry) and the specification that at least four measurements must be included. However, this latter specification, although aimed at increasing the accuracy of the variation calculation, could introduce confounding by indication since patients with more frequent tests might have more severe/less stable disease and hence a higher degree of pathological variation. Furthermore, it has been shown that the ordering of any test by a clinician increases the risk of the disease which the test is investigating, since the presence of a test in a patient record suggests there was a suspicion that the patient was at risk of the disease. This could cause some degree of selection bias when compared with results obtained from healthy populations in controlled biological variation studies.^20^

Assumptions were made for the repeat results calculation, namely that the change between first and second result is representative of changes in the result in subsequent calculations.

Individual sites may have used different devices to conduct their measures, and the nature of THIN does not allow for exploration of this. It was not possible to distinguish between whether the results were prebronchodilator or postbronchodilator or a mixture, but as NICE guidelines do not recommend routine reversibility testing, it is likely that the vast majority of the results are from tests conducted without bronchodilation. Note that as analytical variation is a fixed proportion of the overall variability, it might constitute a relatively larger proportion of the variability in patients with lower lung volumes.

### Interpretation and generalisability

This study shows that the variation of FEV_1_ and FVC in this population of primary care patients in the UK is much higher than results for variation previously reported in the literature. This could be because the participants might be less likely to be stable (although good practice suggests spirometry should only be performed in stable patients), and because the pre-analytical testing conditions were not as rigorously controlled as would be the case in a clinical study. Further, the technical expertise and quality control may be greater in a clinical trial than in practice.^21^ This means that the results of this study are likely to be more generalisable to a primary care population.

The findings of this study have clinical implications for diagnosis and monitoring of respiratory diseases such as COPD.

For diagnosis and monitoring, not taking within-individual variation into account could lead to false positives and the use of inappropriate interventions, while false negatives could lead to appropriate treatment being withheld, delayed diagnosis and increased patient morbidity. This problem could be accentuated when referring to changes from previous results, especially if the variation at the time of measurement is in the opposite direction to the previous measurement.

Minimal clinically important difference (MCID) differs with diagnosis, severity of disease and what is considered clinically important (for example severity of symptoms vs long-term outcomes).^22^ However, a pre-bronchodilator FEV_1_ improvement of around 100 mL is generally considered to correlate with other important clinical outcomes.^23^

The CV_T_ of this study can be used to calculate the probability with which a measured value reflects the true mean of the patient (assuming normal distribution and using a Z-score of difference between means/SD). If we take a hypothetical patient with a true FEV_1_ of 2.75 L the CV_T_ from the data in this study is approximately 18.2%. This suggests a probability of 0.32 that the measured result will be 0.5 L higher or lower than the true mean, which is five times the MCID. For absolute figures for the same patient (SD=2.75 L×18.2%=0.500 L), the RC calculated as *RC=z×√2×SD* (where z=1.96, representing a 95% CI) is 1.39 L, which is nearly 14 times the MCID suggested above.

It has been shown that spirometry results influence decisions regarding medication in the management of COPD and asthma in primary care.^24^ It can therefore be seen that the high variation of between-day spirometry measurements could lead to incorrect treatment decisions being made.

Guidelines for COPD and asthma diagnosis are generally based on prediction equations given as per cent predicted rather than absolute values, but few participants had their FEV_1_ and FVC recorded as per cent predicted in this study, making it hard to make specific estimates for how the variation found in this study would affect clinical outcomes. However, FEV_1_ is used to make clinical judgements regarding individuals as well as regulatory and guideline recommendations,^22^ and it is clear that the high level of between-day within-individual variation seen here would make measurements taken on a single day potentially unrepresentative of a patient’s true status.

Clinicians should therefore be aware of the possibility for misdiagnosis and inappropriate management of COPD when relying on single or small numbers of between-day spirometry measures, and authors of clinical guidelines should take variation into account when making diagnosis, monitoring and treatment recommendations. Guidelines regarding recommended intervals between tests should be amended, and education regarding this issue, especially of primary care providers of spirometry, should be instituted.

Note that some of the underlying diseases for which tests are performed have a higher variability than others, that is pathological variation will comprise a higher proportion of the total variability. For example, asthma is considered a highly variable disease, while well-controlled COPD may have much lower variability.

Nevertheless, these findings show that misclassification of respiratory disease status based on FEV_1_ and FVC measurements due to within-individual variation may be more common than previously thought. However, new Global Initiative for Obstructive Lung Disease guidelines use the ABE classification which does not rely on FEV_1_. There is also a general move to treating endotypes not severity by FEV_1_ nowadays. The variation found in this study would be important if thinking about any therapy that uses an FEV_1_ threshold for decision making.

Further research that addresses confounding by a change in frequency or timing of testing with increased disease severity or variation would be useful. Studies into how variation in this population affects clinical outcomes such as mortality and whether reducing variation with improved testing and management protocols improves outcomes is also important.

## Conclusions

This study suggests that the real-world within-individual total measured variation of the spirometry measures FEV_1_ and FVC are much higher than previously reported in the literature, and that within-individual variation increases with decreasing FEV_1_ and FVC, that is with increasing disease severity. However, since this cohort is selected to have more frequent repeat testing, it may be more unstable than the wider population, limiting generalisability.

## Supplementary material

10.1136/bmjresp-2025-003853 online supplemental file 1

## Data Availability

Data may be obtained from a third party and are not publicly available.
