# Supplementary material for: Within-individual variation of spirometry measurements in primary care: a retrospective cohort study
Source: BMJ Open Respir Res. 2026 May 17;13(1):e003853. doi: 10.1136/bmjresp-2025-003853 (PMC13182296; doi:10.1136/bmjresp-2025-003853)
Supplement: online supplemental file 1 [file bmjresp-13-1-s001.pdf]

Supplementary Information

AUTHOR CONTRIBUTIONS

Conceptualization, AG, TM, AS

Data curation, AG

Formal analysis, AG

Investigation, AG

Methodology, AG, TM, AS, AT

Project administration, AG

Supervision, TM, AS

Writing – original draft, AG

Writing – review & editing, AG, TM, AS, AT

AG acted as guarantor

ORCID

Alex Gough (https://orcid.org/0000-0001-6954-7408)

Tom Marshall 0000-0001-9277-5214

Alice Sitch https://orcid.org/0000-0001-7727-4497

Alice Turner 0000-0002-5947-3254

DATA AVAILABILITY

Data cannot be shared publicly because the authors do not have permission to share the data. IMRD data used for the study were obtained under licence from IQVIA; pseudonymised participant data are available from IQVIA subject to Scientific Review Committee approval. IMRD-UK data contains electronic health records from UK primary care. In compliance with the UK Data Protection Act and licensing agreements, the data cannot be shared via a public repository. These restrictions aim to protect patient confidentiality. The data underlying the results presented in the study are available from IQVIA (https://www.iqvia.com/locations/united-kingdom/solutions/life-sciences-industry solutions/real-world-solutions/iqvia-medical-research-data)

Appendix 1 - Subgroup analysis

Table S1a Subgroup analyses for FEV1. Total N=4412

		N	%	Mean	CV (95%CI)	95% LCI	95% UCI
Comorbidities	No comorbidity	229	5.2	2.73	13.5	12.7	14.3
	Hypertension	1,417	32.1	1.83	24.3	23.6	24.9
	Asthma	1,805	40.9	2.05	21.6	21	22.1
	COPD	1,514	34.3	1.72	29.1	28.4	29.9
	Hyperthyroidism	78	1.8	1.67	21.8	19.4	24.3

		N	%	Mean	CV (95%CI)	95% LCI	95% UCI
	Hypothyroidism	280	6.3	1.77	20.9	19.6	22.1
	CKD stage 3 to 5	245	5.6	1.72	23.7	22.2	25.1
	Heart failure	112	2.5	1.72	29.1	26.2	31.9
	Ischaemic heart disease	563	12.8	1.83	23.4	22.4	24.4
	Ischaemic stroke	50	1.1	1.76	24.1	20.7	27.5
	Haemorrhagic Stroke	20	0.5	x	x	x	x
	Cancer	236	5.3	1.72	24.9	23.2	26.5
	DM or Pre DM	19	0.4	x	x	x	x
Deprivation score	1	747	16.9	2.11	20.2	19.4	21
	2	786	17.8	2.04	21.2	20.4	22
	3	904	20.5	2.04	23.6	22.7	24.4
	4	923	20.9	1.99	23.6	22.8	24.4
	5	598	13.6	1.96	23.9	22.8	24.9
	Missing	454	10.3	1.99	21.9	20.8	23
Region	East Midlands	72	1.6	2.12	16.8	14.4	19.1
	East of England	283	6.4	2.02	18.8	17.6	20
	London	390	8.8	1.99	19.9	18.8	21
	North East	120	2.7	1.83	25.1	22.6	27.7
	North West	552	12.5	1.96	24.4	23.3	25.5
	Northern Ireland	168	3.8	2.16	21.7	20	23.3
	Scotland	451	10.2	2.04	22.5	21.4	23.6
	South Central	371	8.4	2.11	21.2	20	22.3
	South East Coast	372	8.4	2.06	21.6	20.5	22.8
	South West	264	6.0	1.99	23.1	21.7	24.6
	Wales	482	10.9	1.99	21.3	20.3	22.3
	West Midlands	692	15.7	2.04	25.5	24.5	26.5
	Yorkshire & Humber	195	4.4	2.08	20.7	19.1	22.4
Alcohol consumption	Current drinker	2,775	62.9	2.05	22.7	22.2	23.1
	Ex-drinker	71	1.6	2.09	25.3	22.3	28.2
	Teetotal	610	13.8	1.83	24.5	23.5	25.6
	Missing	956	21.7	X	X	X	X

Table S1b Subgroup analysis for FVC. Total N=3567

		N	%	Mean	CV % (95%CI)	95% LCI	95% UCI
Comorbidities	No comorbidity	201	5.6	3.59	12	11.2	12.7
	Hypertension	1,131	31.7	2.79	16.5	16.1	17
	Asthma	1,422	39.9	3.08	14.9	14.5	15.3
	COPD	1,087	30.5	2.80	17.5	16.9	18
	Hyperthyroidism	61	1.7	2.55	16.5	14.6	18.5
	Hypothyroidism	245	6.9	2.62	17.5	16.5	18.6
	CKD stage 3 to 5	211	5.9	2.59	17.7	16.6	18.8
	Heart failure	90	2.5	2.75	21.7	19.5	24
	Ischaemic heart disease	405	11.4	2.80	18.3	17.4	19.1
	Ischaemic stroke	39	1.1	x	x	x	x
	Haemorrhagic Stroke	17	0.5	x	x	x	x
	Cancer	198	5.6	2.73	17.6	16.4	18.7
	DM or preDM	14	0.4	x	x	x	x
Deprivation score	1	658	18.4	3.08	14.8	14.2	15.4
	2	600	16.8	3.09	14.3	13.8	14.9
	3	790	22.1	3.05	15.9	15.3	16.4
	4	711	19.9	2.96	16.2	15.6	16.8
	5	435	12.2	2.95	14.8	14.1	15.5
	Missing	373	10.5	3.06	14.6	13.9	15.3
Region	East Midlands	55	1.5	3.16	10.4	8.9	11.9
	East of England	266	7.5	3.02	14.6	13.7	15.4
	London	222	6.2	2.92	16.4	15.3	17.5
	North East	71	2.0	2.53	14.6	12.7	16.5
	North West	366	10.3	2.86	16	15.2	16.8
	Northern Ireland	154	4.3	3.30	13.5	12.5	14.6
	Scotland	347	9.7	3.16	16.7	15.9	17.6
	South Central	331	9.3	3.17	12.7	12	13.4

		N	%	Mean	CV % (95%CI)	95% LCI	95% UCI
	South East Coast	429	12.0	2.98	13.7	13.1	14.4
	South West	201	5.6	3.07	12.2	11.4	13.1
	Wales	450	12.6	3.14	15.5	14.8	16.1
	West Midlands	493	13.8	2.89	17.7	16.9	18.5
	Yorkshire & Humber	182	5.1	3.13	15.6	14.4	16.7
Alcohol consumption	Current drinker	2,200	61.7	3.07	15.2	14.9	15.5
	Ex drinker	61	1.7	3.04	16	14.2	17.9
	Teetotal	495	13.9	2.68	16.7	15.9	17.4
	Missing	811	22.7	-	-	-	-

Table S2a Multiple subgroup analysis for FEV1

Sex	Age	Resp status C=COPD, A=Asthma, N=no resp disease	Never smoked =NS, Smoker or exsmoker = S	BMI		CV(%)	N
	Y=20 to 60; O=60+			L<30=L >30=H			
M	Y	N	NS	L		15.2	25
M	Y	N	NS	H		x	8
M	Y	N	S	L		14.1	32
M	Y	N	S	H		17.3	11
F	Y	N	NS	L		17.8	29
F	Y	N	NS	H		x	6
F	Y	N	S	L		10.8	27
F	Y	N	S	H		13.4	12
M	O	N	NS	L		15.3	42
M	O	N	NS	H		x	8
M	O	N	S	L		16.6	55
M	O	N	S	H		19.3	15
F	O	N	NS	L		14.2	46
F	O	N	NS	H		15.5	25
F	O	N	S	L		12.1	43
F	O	N	S	H		11.5	15
M	Y	A	NS	L		16	59

M	Y	A	NS	H		x	6
M	Y	A	S	L		23.5	77
M	Y	A	S	H		24.3	26
F	Y	A	NS	L		15.4	70
F	Y	A	NS	H		13.7	21
F	Y	A	S	L		22.3	83
F	Y	A	S	H		19.5	33
M	O	A	NS	L		20.9	125
M	O	A	NS	H		20.4	28
M	O	A	S	L		25.5	280
M	O	A	S	H		20.7	75
F	O	A	NS	L		19.5	156
F	O	A	NS	H		19.2	57
F	O	A	S	L		24.9	221
F	O	A	S	H		22.9	83
M	Y	C	NS	L		x	3
M	Y	C	NS	H		x	0
M	Y	C	S	L		32.7	39
M	Y	C	S	H		25.8	10
F	Y	C	NS	L		x	5
F	Y	C	NS	H		x	0
F	Y	C	S	L		29	41
F	Y	C	S	H		23.1	15
M	O	C	NS	L		31.3	73
M	O	C	NS	H		31.7	16
M	O	C	S	L		30.5	416
M	O	C	S	H		25.2	97
F	O	C	NS	L		29.2	75
F	O	C	NS	H		21.7	17

F	O	C	S	L		27.7	311
F	O	C	S	H		24.4	68

Table S2b Multiple subgroup analysis for FVC

Sex	Age	Resp status C=COPD, A=Asthma, N=no resp disease	Never smoked =NS, Smoker or exsmoker = S	BMI		N		CV (%)
	Y=20 to 60; O=60+			L<30=L >30=H				
M	Y	N	NS	L		28		10
M	Y	N	NS	H		7		x
M	Y	N	S	L		20		7.9
M	Y	N	S	H		7		x
F	Y	N	NS	L		30		19.1
F	Y	N	NS	H		6		x
F	Y	N	S	L		18		12.4
F	Y	N	S	H		10		14.9
M	O	N	NS	L		45		15.5
M	O	N	NS	H		9		x
M	O	N	S	L		47		12.5
M	O	N	S	H		15		12.8
F	O	N	NS	L		43		11.4
F	O	N	NS	H		26		13.8

F	O	N	S	L		33		25.9
F	O	N	S	H		20		11.1
M	Y	A	NS	L		60		11.5
M	Y	A	NS	H		13		10.2
M	Y	A	S	L		69		13.6
M	Y	A	S	H		26		18.2
F	Y	A	NS	L		68		12.9
F	Y	A	NS	H		20		9
F	Y	A	S	L		63		12.9
F	Y	A	S	H		32		17.7
M	O	A	NS	L		95		14.8
M	O	A	NS	H		21		16.4
M	O	A	S	L		219		16.4
M	O	A	S	H		58		14.8
F	O	A	NS	L		114		18.1
F	O	A	NS	H		44		11.4
F	O	A	S	L		155		16.6
F	O	A	S	H		59		15.3
M	Y	C	NS	L		3		x
M	Y	C	NS	H		0		x
M	Y	C	S	L		34		17.9
M	Y	C	S	H		8		x
F	Y	C	NS	L		4		x
F	Y	C	NS	H		0		x
F	Y	C	S	L		31		16.5
F	Y	C	S	H		9		x
M	O	C	NS	L		46		19.6
M	O	C	NS	H		14		17.6
M	O	C	S	L		290		17.1

M	O	C	S	H		73		17
F	O	C	NS	L		40		19.6
F	O	C	NS	H		14		12.5
F	O	C	S	L		221		16.8
F	O	C	S	H		46		16.1

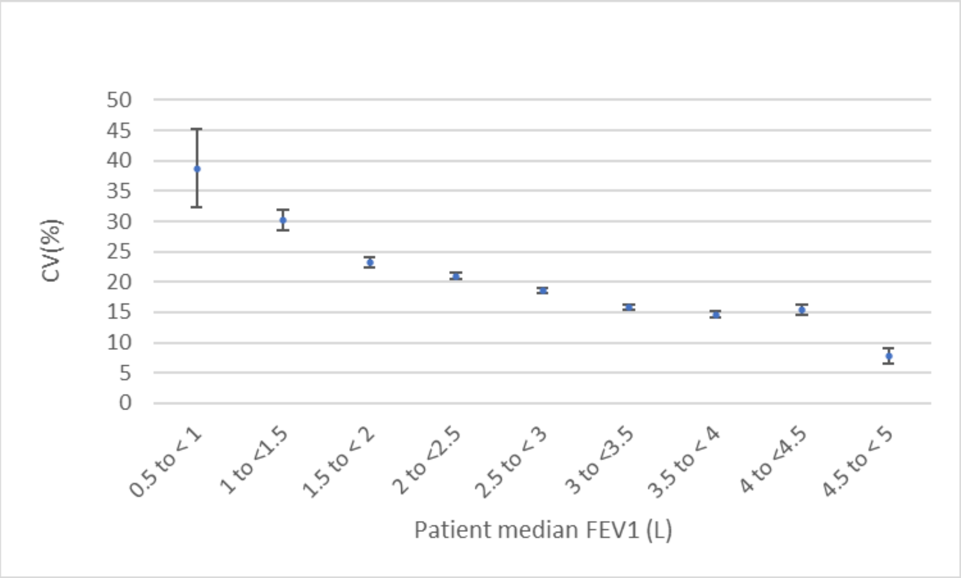

Figure S1a Coefficient of variation of FEV_1 by patient median with 95% confidence intervals. Median FEV_1 is in L. Total N=4412

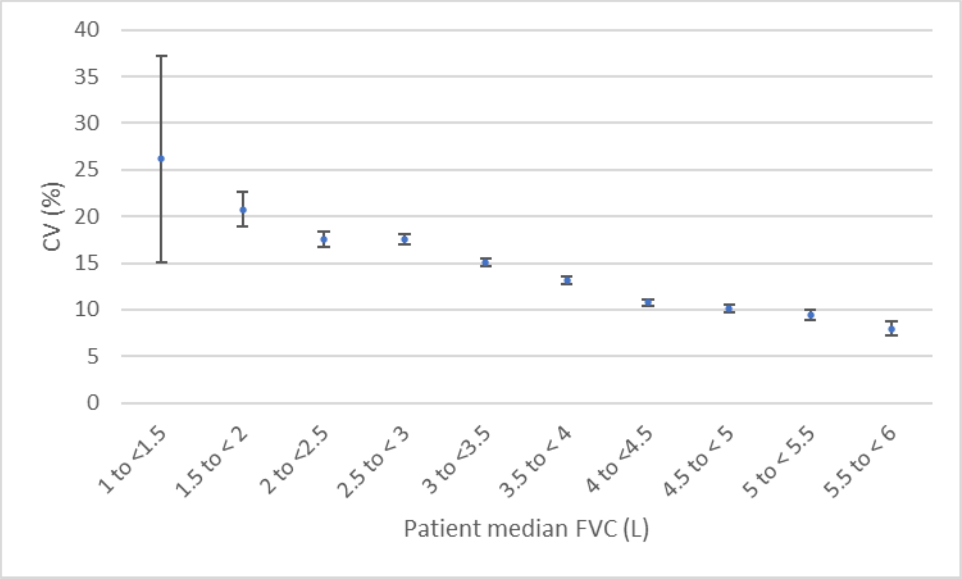

Figure S1b Coefficient of variation of FVC by patient median with 95% confidence intervals. Median FVC is in L.

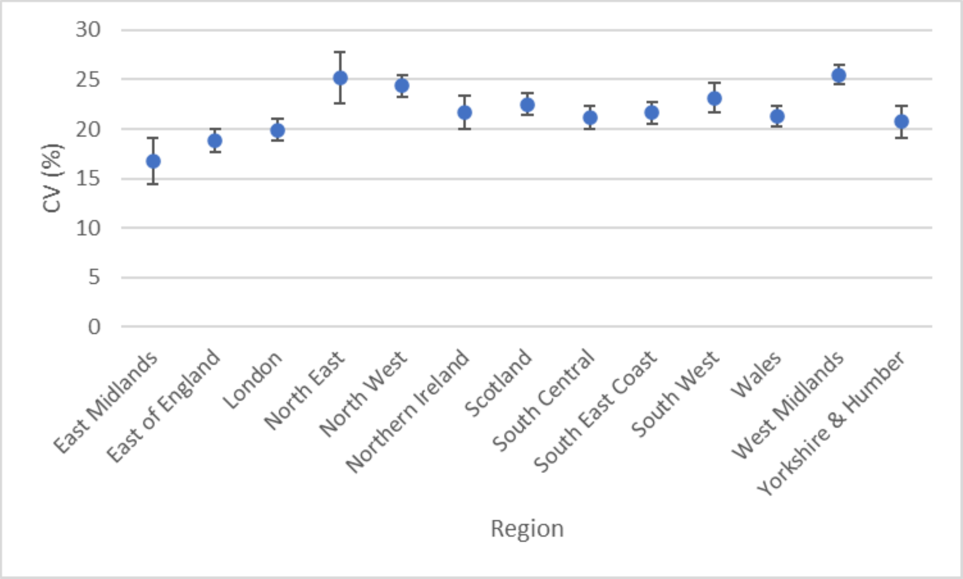

Figure S2 CVT by region for FEV1

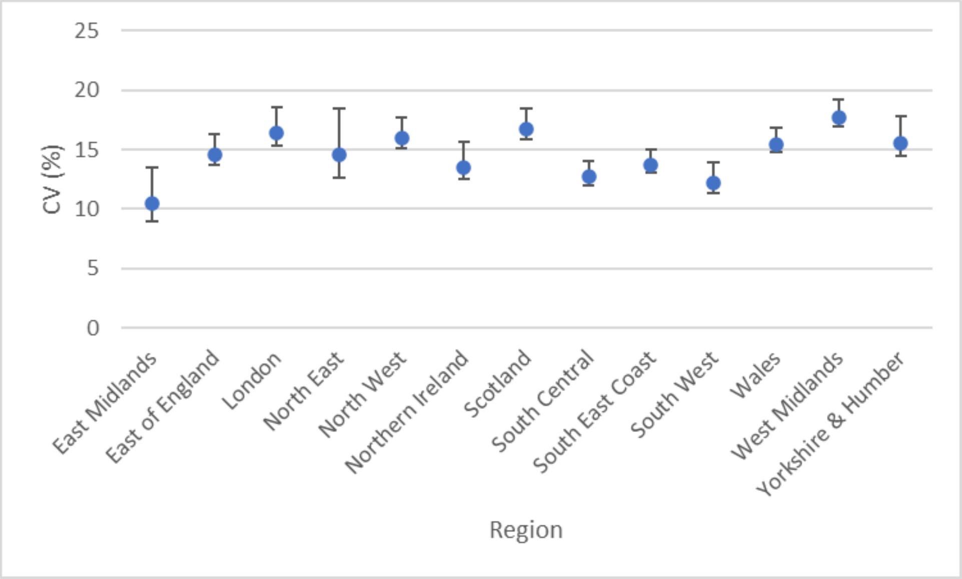

Figure S3 CVT by region for FVC

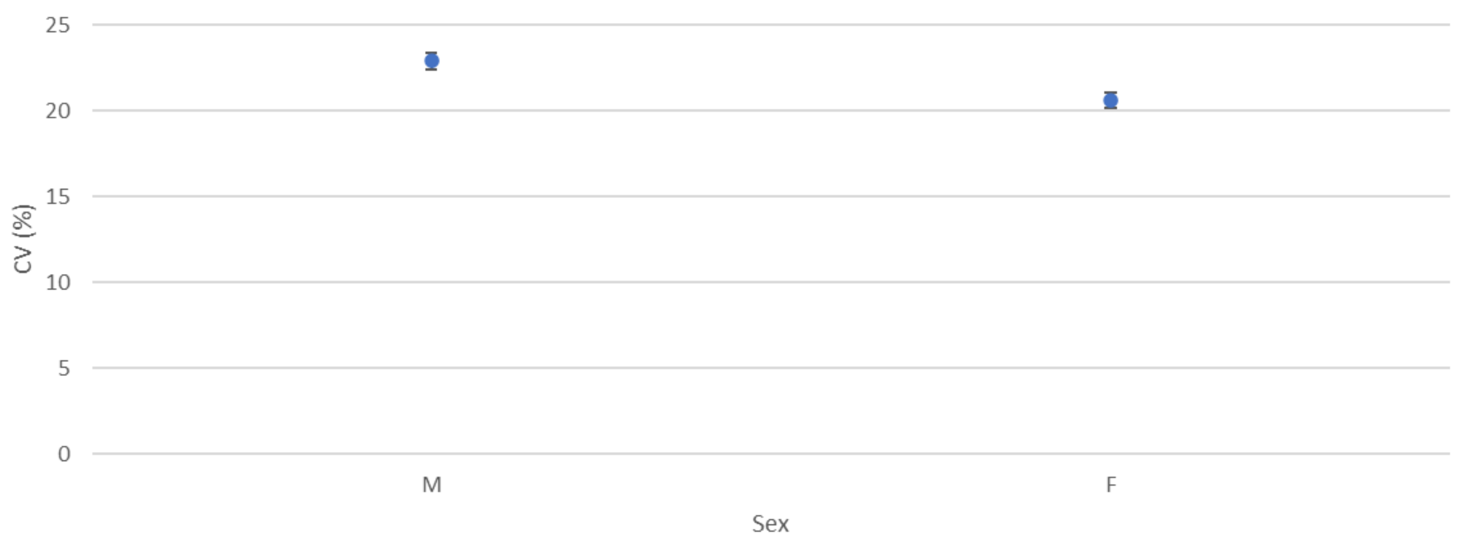

Figure S4 CVT by sex for FEV1

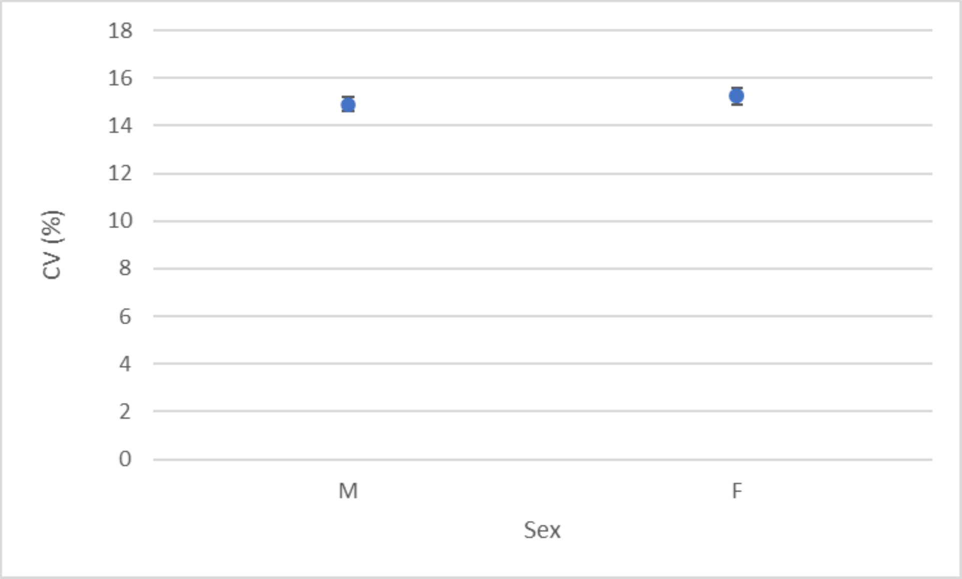

Figure S5 CVT by sex for FVC

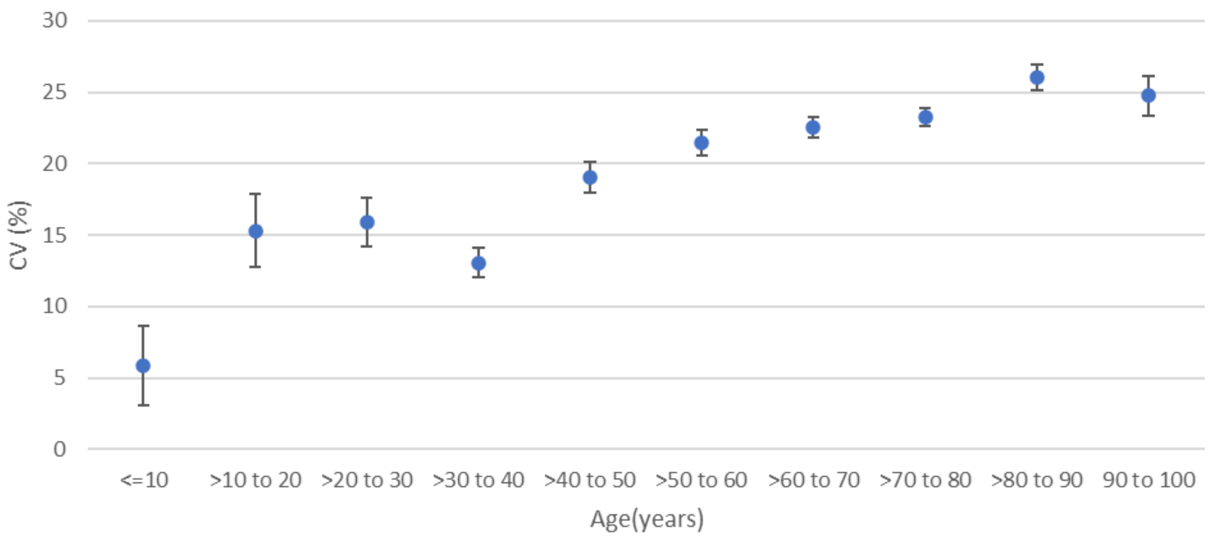

Figure S6 CVT by age for FEV1

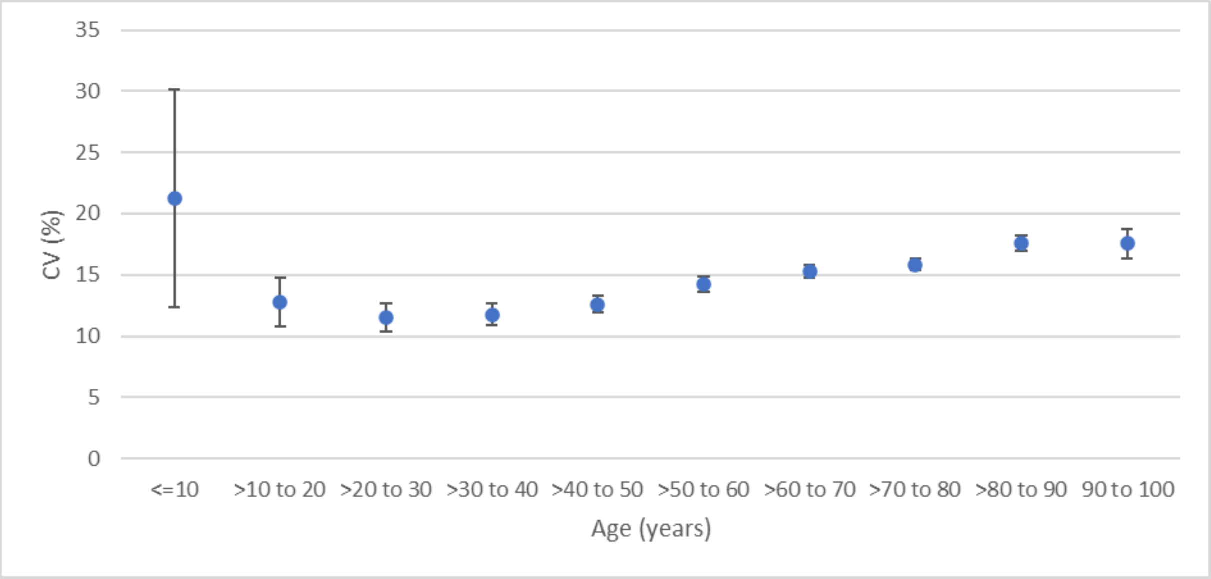

Figure S7 CVT by age for FVC

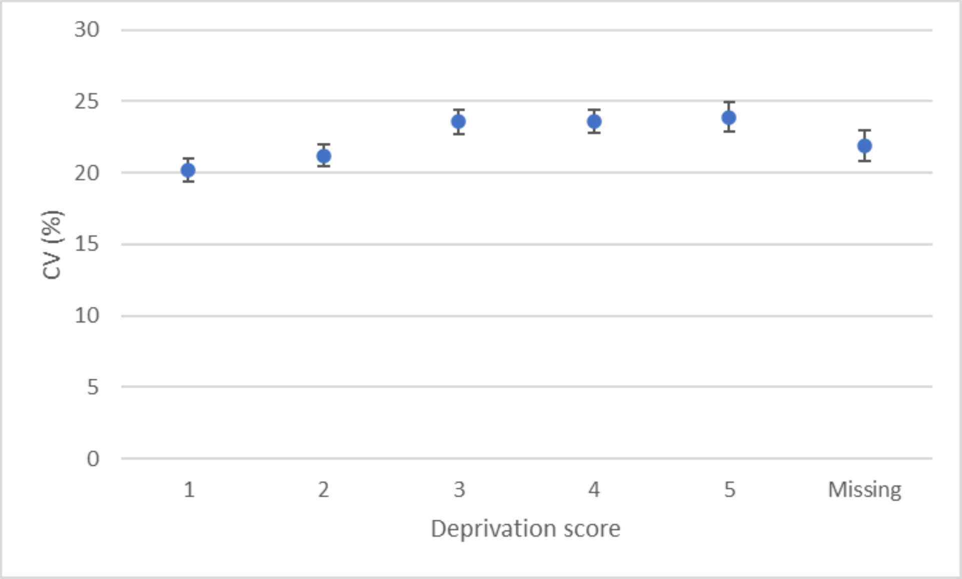

Figure S8 CVT by Townsend deprivation score for FEV1

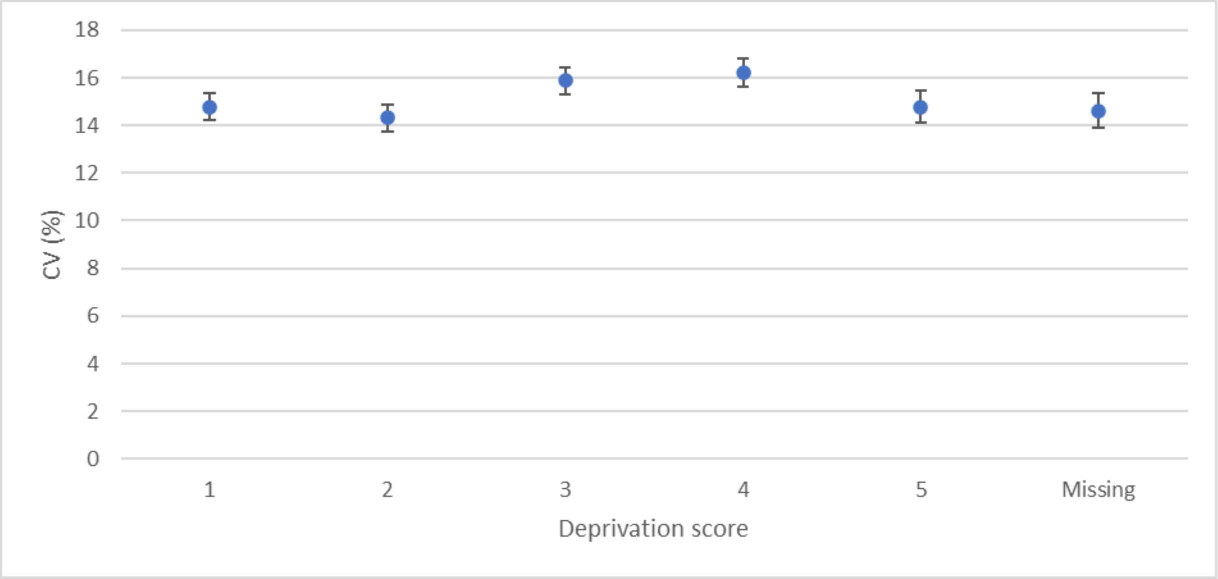

Figure S9 CVT by Townsend deprivation score for FVC

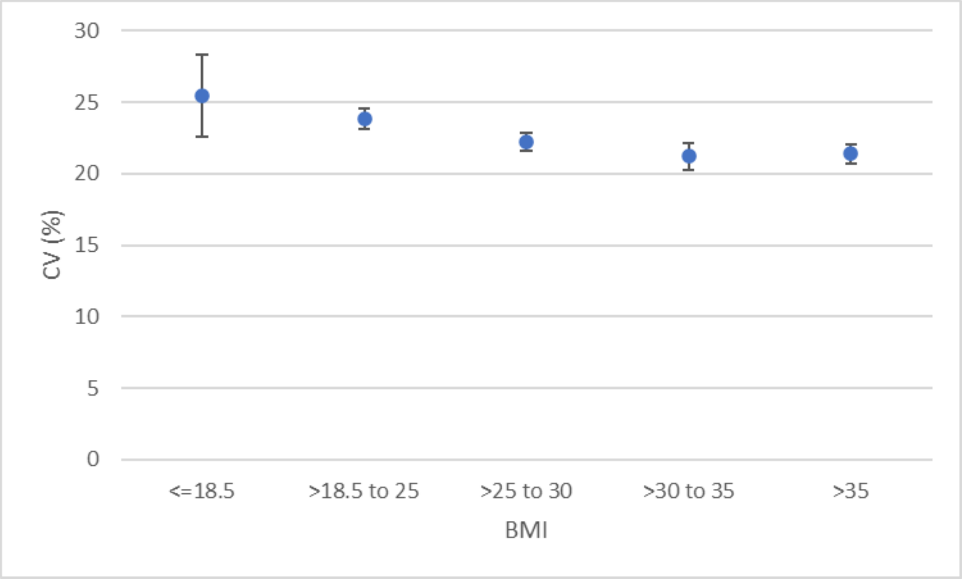

Figure S10 CVT by BMI for FEV1

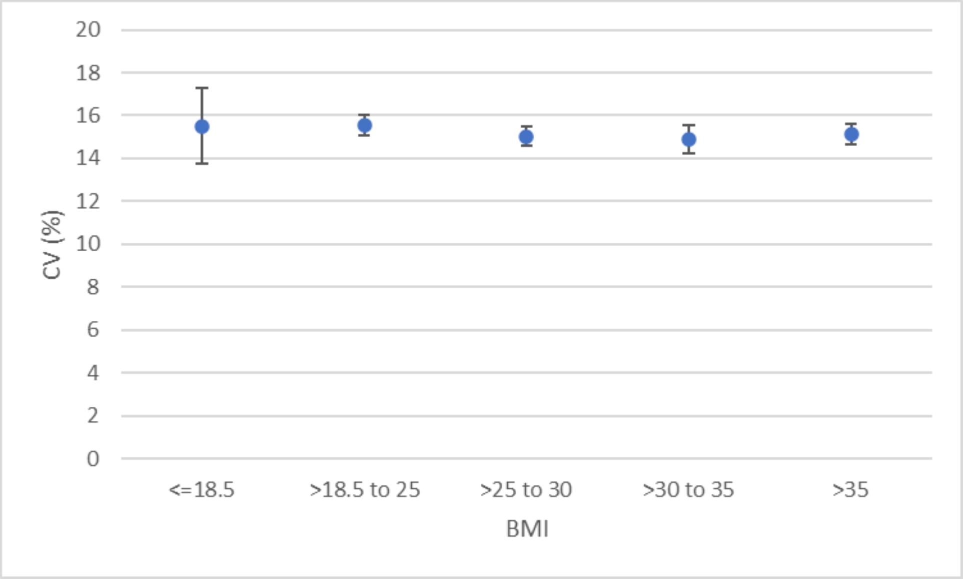

Figure S11 CVT by BMI for FVC

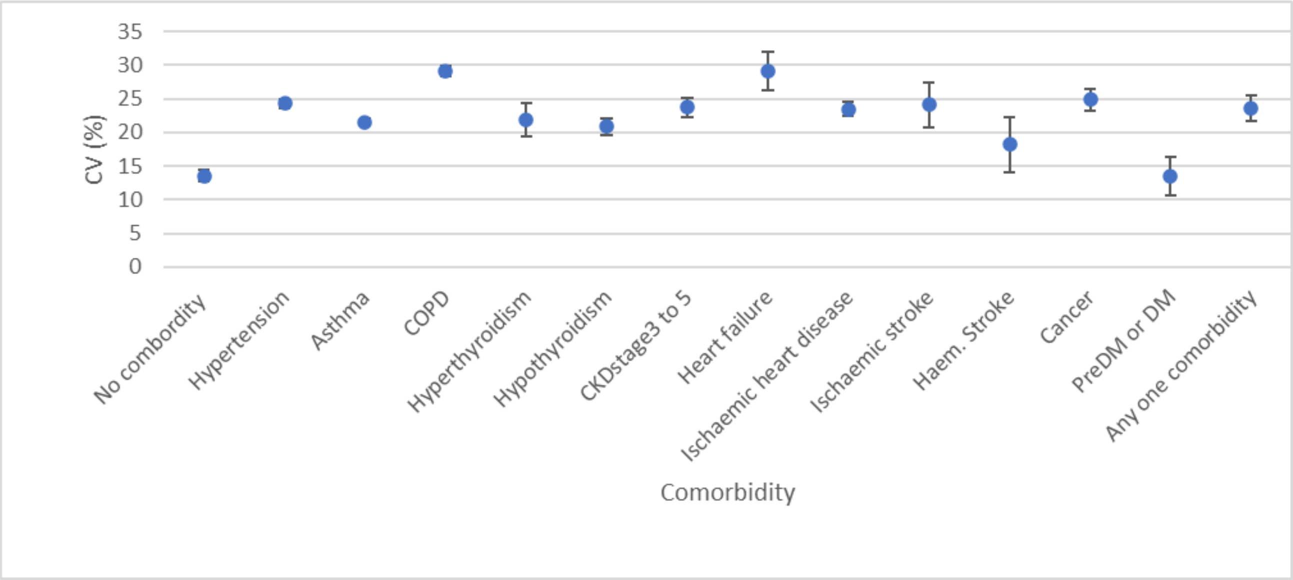

Figure S12 CVT by comorbidities for FEV1

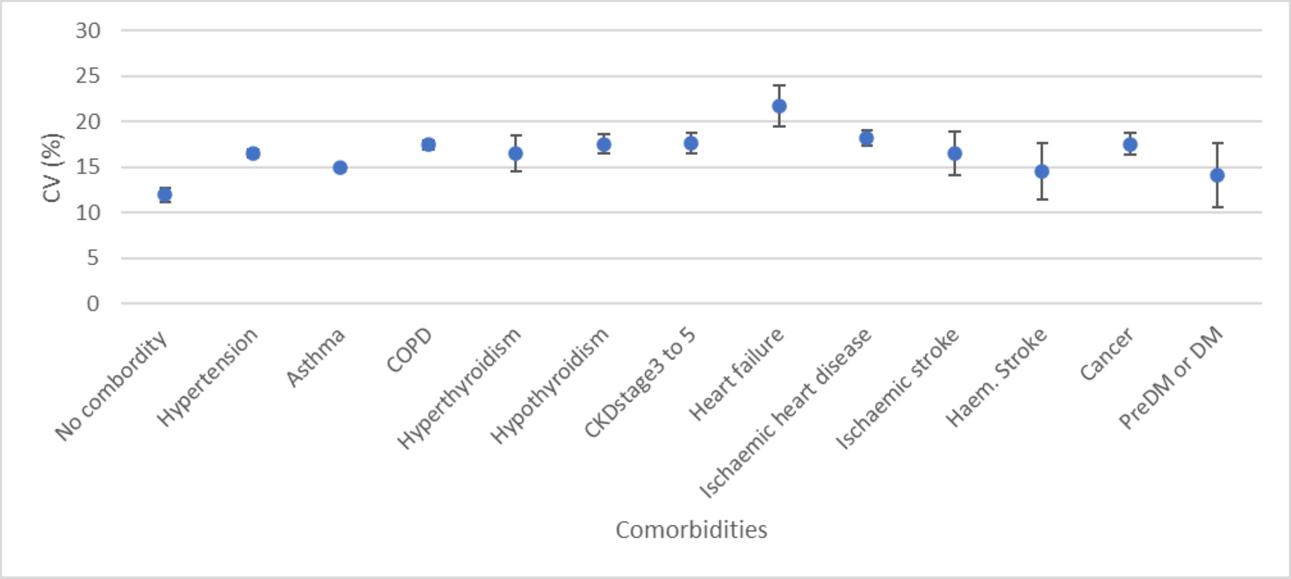

Figure S13 CVT by comorbidities for FVC

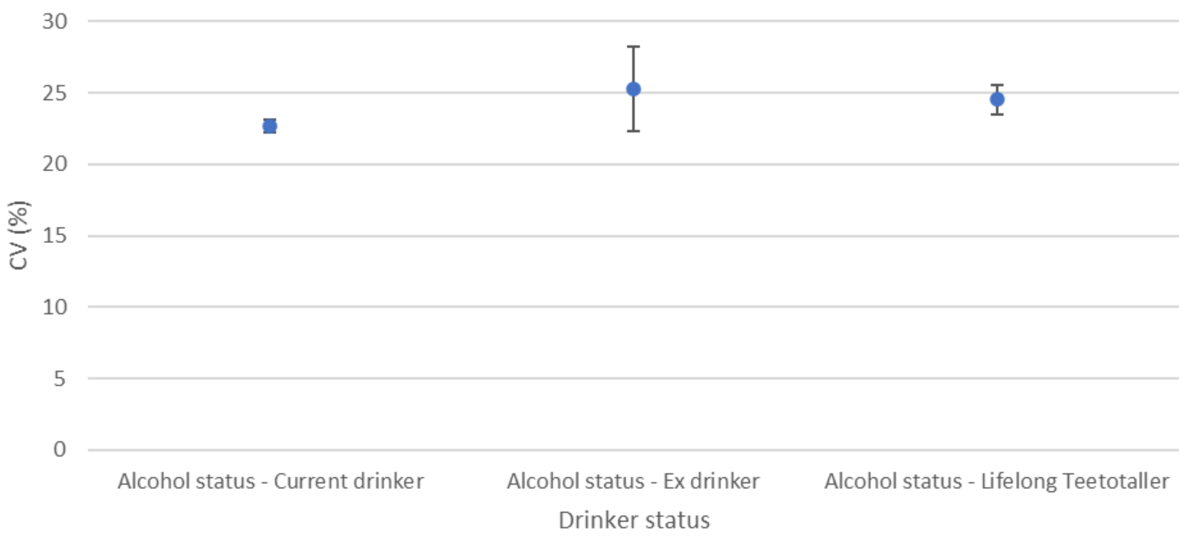

Figure S14 CVT by alcohol consumption for FEV1

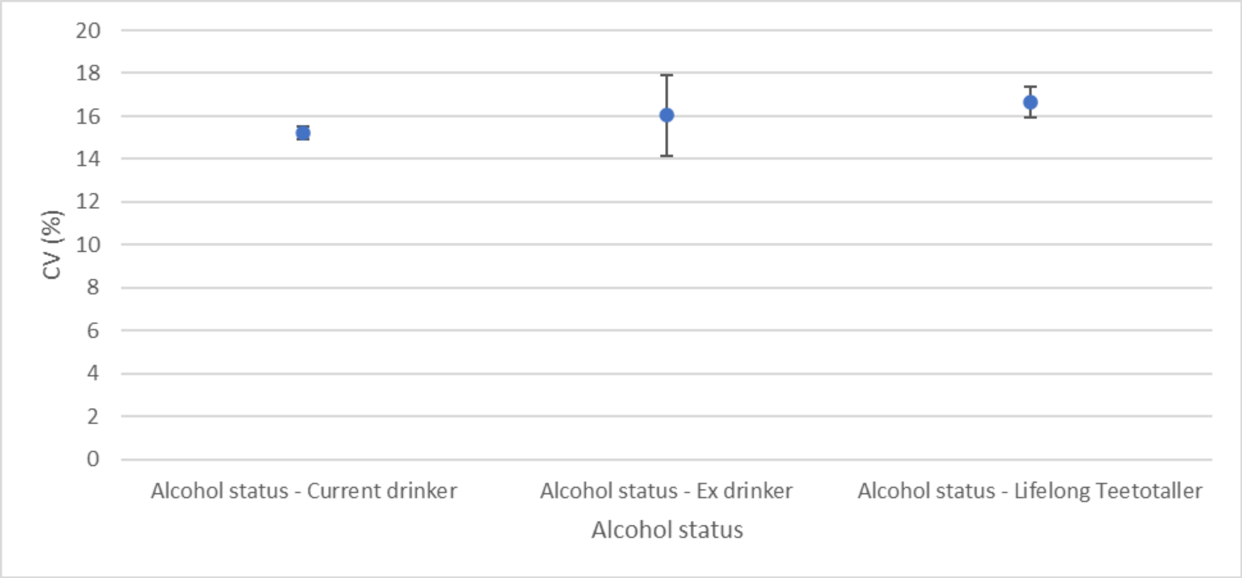

Figure S15 CVT by alcohol consumption for FVC

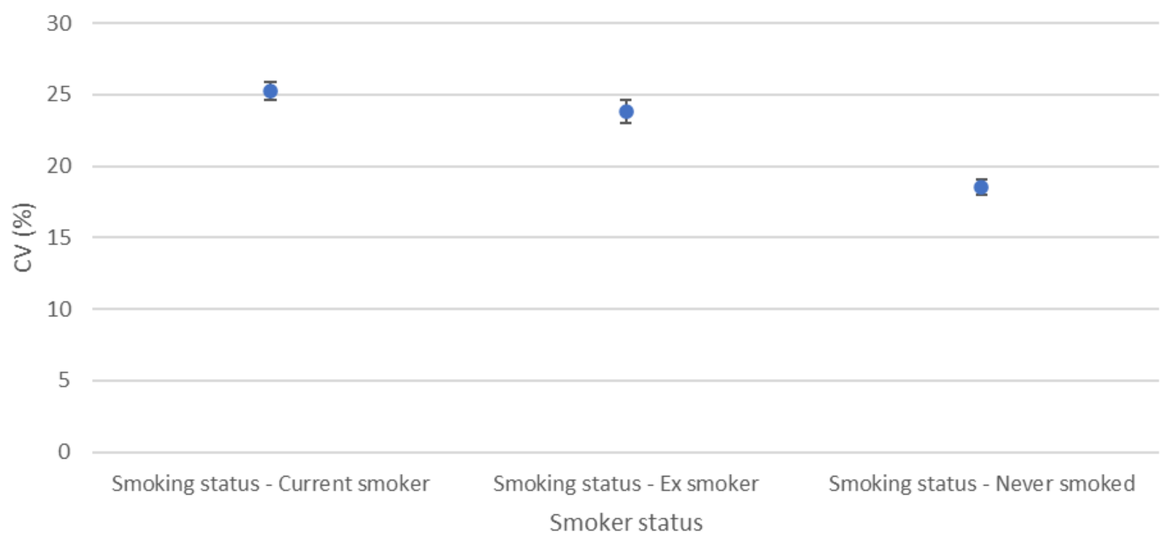

Figure S16 CVT by smoker status for FEV1

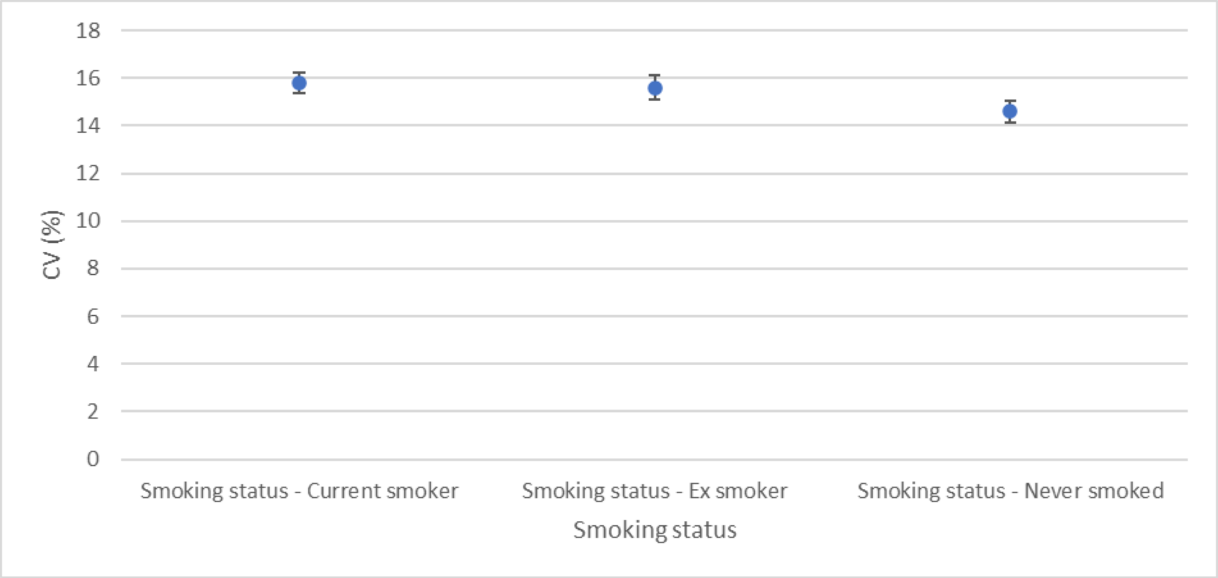

Figure S17 CVT by smoker status for FVC

Appendix 2 – Sensitivity analyses

Table S3a Sensitivity analyses for FEV1

**=range. Total N=4412 for denominator for %.*

		N	%	Mean	CV (95%CI)	95% LCI	95% UCI
Year	2010	164	N/A	1.93	17.3	15.9	18.7
	2011	201	N/A	2.09	22.5	20.8	24.1
	2012	279	N/A	2.15	21.7	20.3	23
	2013	251	N/A	2.31	20.4	19.1	21.7
	2014	197	N/A	2.31	20.2	18.8	21.7
	2015	155	N/A	2.20	23.9	22	25.8
	2016	145	N/A	2.23	27.2	25	29.4
	2017	108	N/A	2.16	27.1	24.5	29.7
	2018	103	N/A	2.35	23.7	21.3	26
	2019	76	N/A	2.44	21.2	18.7	23.6
Quarter	1	376	N/A	2.12	24.7	23.5	26
	2	352	N/A	2.17	23	21.8	24.2
	3	314	N/A	2.14	23.7	22.3	25.1
	4	352	N/A	2.15	22.8	21.6	24.1
Number of measurements	2	57,572	N/A	1.97	19.1	19	19.2
	3	11,476	N/A	1.95	20.8	20.5	21
	4	3,284	N/A	2.03	22.2	21.8	22.6
	5	681	N/A	1.96	23.1	22.2	24
	6	262	N/A	2.10	22.6	21.3	23.9
	7	64	N/A	2.12	21.5	18.8	24.2

		N	%	Mean	CV (95%CI)	95% LCI	95% UCI
Patient median FEV1	0 to <0.5	9	0.2	-	-	-	-
	0.5 to < 1	348	7.9	0.88	38.7	37	40.5
	1 to <1.5	854	19.4	1.31	30.2	29.4	31
	1.5 to < 2	1,124	25.5	1.77	23.2	22.7	23.7
	2 to <2.5	962	21.8	2.22	21	20.5	21.4
	2.5 to < 3	575	13.0	2.68	18.6	18.1	19.1
	3 to <3.5	314	7.1	3.16	15.8	15.2	16.4
	3.5 to < 4	155	3.5	3.64	14.7	13.8	15.5
	4 to <4.5	58	1.3	4.08	15.3	14	16.7
Days between measurements	0 to < 4	1,503	34.1	2.26	25.6	25	26.2
	4 to < 7	88	2.0	2.22	23.5	20.8	26.1
	7 to < 14	399	9.0	1.97	23	21.9	24.2
	14 to < 21	498	11.3	1.94	20.6	19.6	21.5
	21 to < 28	458	10.4	1.89	17.4	16.5	18.3
	28 to < 60	1,341	30.4	1.87	17.9	17.4	18.5
	60 to < 90	125	2.8	1.78	21.8	19.6	24
Method	Linear regression	4412	100	2.03	22.4	22.1	22.8
	Arithmetic	4412	100	2.03	018.1	0.2 (lower range)	158.5 (upper range)
Unit	L	4412	100	2.03	22.4	22.1	22.8
	% predicted	515	N/A	66.97	16.0	15.4	16.7

Table S3b Sensitivity analyses for FVC.

Total N=3567 for denominator for %.

		N	%	Mean	CV	95% LCI	95% UCI
Year	2010	101	N/A	2.95	16.6	15	18.2
	2011	191	N/A	3.06	13.8	12.8	14.8
	2012	230	N/A	3.09	14.4	13.6	15.3
	2013	211	N/A	3.21	11.9	11.2	12.7
	2014	185	N/A	3.25	13.8	12.8	14.8
	2015	147	N/A	3.21	14.4	13.3	15.5
	2016	161	N/A	3.30	18.3	17	19.7
	2017	141	N/A	3.22	15	13.8	16.1
	2018	154	N/A	3.23	15.4	14.3	16.6
	2019	149	N/A	3.33	15.8	14.6	17
Quarter	1	359	N/A	3.08	16.5	15.7	17.3
	2	331	N/A	3.11	15.6	14.8	16.4
	3	332	N/A	3.07	13.8	13.1	14.6
	4	319	N/A	3.17	15.4	14.6	16.3
Number of measurements	2	40,301	N/A	2.90	13.8	13.7	13.9
	3	7,772	N/A	2.91	14.6	14.4	14.8
	4	2,488	N/A	3.00	15.2	14.9	15.6
	5	622	N/A	3.08	15	14.5	15.6
	6	278	N/A	3.15	15.2	14.5	16
	7	68	N/A	3.25	15.5	13.9	17.1
Patient median FVC	0 to <0.5	0	0	-	-	-	-
	0.5 to < 1	22	0.6	-	-	-	-
	1 to <1.5	123	3.4	1.36	26.2	24.3	28
	1.5 to < 2	366	10.3	1.82	20.8	19.9	21.6

		N	%	Mean	CV	95% LCI	95% UCI
	2 to <2.5	656	18.4	2.26	17.5	17	18
	2.5 to < 3	680	19.1	2.74	17.5	17.1	18
	3 to <3.5	621	17.4	3.22	15.1	14.7	15.5
	3.5 to < 4	490	13.7	3.70	13.1	12.7	13.5
	4 to <4.5	315	8.8	4.20	10.7	10.3	11.2
	4.5 to < 5	158	4.4	4.65	10.2	9.6	10.7
	5 to < 5.5	89	2.5	5.18	9.4	8.7	10.1
Days between measurements	0 to < 4	1,784	50.0	3.18	16.1	15.8	16.5
	4 to < 7	63	1.8	2.85	16.2	14.4	18.1
	7 to < 14	265	7.4	2.97	15	14.1	15.8
	14 to < 21	297	8.3	2.85	14.5	13.7	15.3
	21 to < 28	279	7.8	2.86	13.3	12.5	14.1
	28 to < 60	808	22.7	2.88	13.3	12.8	13.7
	60 to < 90	71	2.0	2.77	12.5	10.9	14
Method	Linear regression	3,567	100	3.03	15.2	15	15.5
	Arithmetic	3567	100	3.04	13.1	0 (lower range)	80.5 (upper range)
Unit	L	3,567	100	3.03	15.2	15	15.5
	% predicted	100	N/A	81.03	12.8	11.6	14

Table S4a Results for long-term data (i.e. all measurements included) for FEV1. Total N=129,161 for denominator for %.

		N	%	Mean	CV	95% LCI	95% UCI
All		129,161	100	1.72	22.8	22.7	22.9
Sex	Male	67,332	52.1	1.96	22.8	22.7	22.9
	Female	61,829	47.9	1.46	21.9	21.8	22
Age	11 to 20	384	0.3	2.21	23.4	22.4	24.4
	21 to 30	861	0.7	2.77	19	18.5	19.6
	31 to 40	1,120	0.9	2.96	15	14.7	15.4
	41 to 50	3,169	2.5	2.66	17.9	17.6	18.1
	51 to 60	10,828	8.4	2.26	20.2	20.1	20.4
	61 to 70	24,690	19.1	1.91	22.5	22.4	22.6
	71 to 80	41,666	32.3	1.68	23.3	23.2	23.4
	81 to 90	33,762	26.1	1.46	23.9	23.8	24
	91 to 100	12,049	9.3	1.27	25.2	25	25.4
BMI	<18.5	3,100	2.4	1.46	27.9	27.3	28.4
	18.5 to <25	41,225	31.9	1.66	24.1	24	24.2
	25 to <30	37,338	28.9	1.76	21.8	21.7	21.9
	30 to <35	15,867	12.3	1.77	21.6	21.4	21.7
	>35	7,689	6.0	1.71	21	20.7	21.2
	Missing	23,942	18.5	1.77	23	22.8	23.1
Smoker status	Current smoker	54,790	42.4	1.69	24.2	24.1	24.3
	Ex-smoker	36,448	28.2	1.65	22.7	22.5	22.8
	Never smoked	24,364	18.9	1.83	20	19.9	20.2
	Missing	13,559	10.5		0	0	0
Respiratory status	Asthma	68,964	53.4	1.71	22.9	22.8	23
	COPD	99,609	77.1	1.58	24.9	24.8	25

Table S4b Results for long-term data (i.e. all measurements included) for FVC. Total N=75,959 for denominator for %.

		N	%	Mean	CV	95% LCI	95% UCI
All		75,956	100	2.73	16.4	16.4	16.5
Sex	Male	39,722	52.3	3.18	16.2	16.1	16.3
	Female	36,234	47.7	2.23	16	16	16.1
Age	11 to 20	338	0.4	2.63	23.7	22.6	24.8
	21 to 30	725	1.0	3.44	18	17.5	18.6
	31 to 40	935	1.2	3.86	11.9	11.6	12.3
	41 to 50	2,244	3.0	3.64	13.2	13	13.5
	51 to 60	6,787	8.9	3.30	14.4	14.2	14.5
	61 to 70	14,912	19.6	2.97	15.8	15.7	15.9
	71 to 80	24,546	32.3	2.70	16.4	16.3	16.5
	81 to 90	19,013	25.0	2.40	17.6	17.5	17.7
	91 to 100	6,154	8.1	2.13	19.1	18.9	19.3
BMI	<18.5	1,704	2.2	2.51	17.7	17.3	18.1
	18.5 to <25	24,096	31.7	2.73	16.5	16.4	16.6
	25 to <30	22,061	29.0	2.75	16.2	16.1	16.3
	30 to <35	9,216	12.1	2.66	16.2	16	16.3
	>35	4,460	5.9	2.51	16.8	16.6	17
	Missing	14,419	19.0	2.84	16.5	16.4	16.6
Smoker status	Current smoker	31,188	41.1	2.75	16.6	16.6	16.7
	Ex-smoker	20,625	27.2	2.67	16.5	16.4	16.6
	Never smoked	15,553	20.5	2.71	15.7	15.5	15.8
	Missing	8590	11.3		0	0	0
Respiratory status	Asthma	41,639	54.8	2.70	16.6	16.5	16.7
	COPD	53,824	70.9	2.62	17.1	17.1	17.2

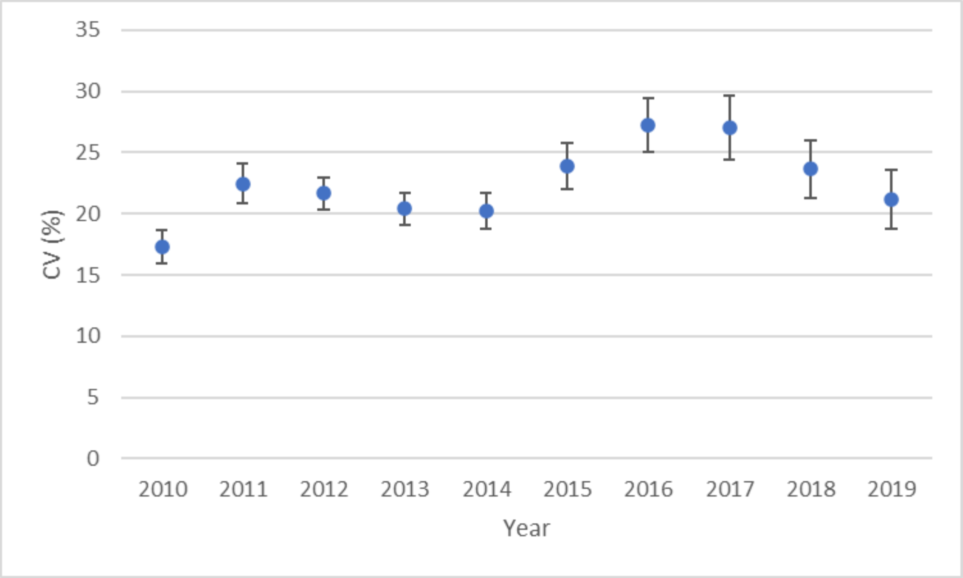

Figure S18 CVT by year for FEV1

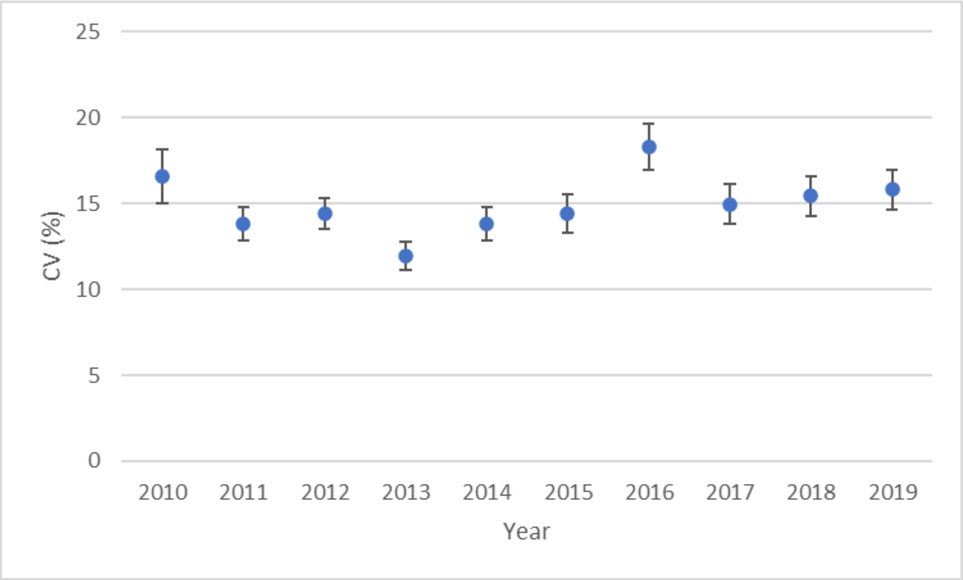

Figure S19 CVT by year for FVC

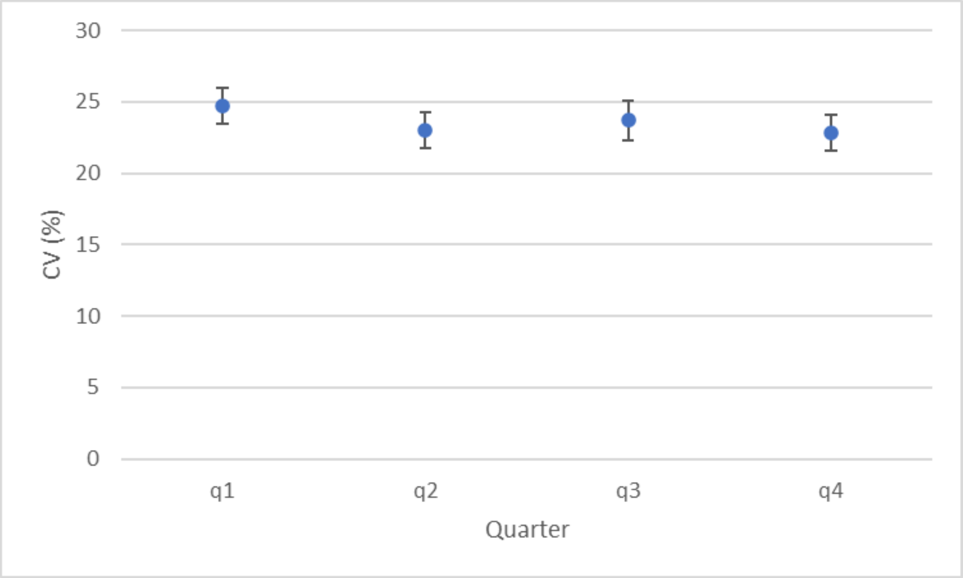

Figure S20 CVT by quarter for FEV1

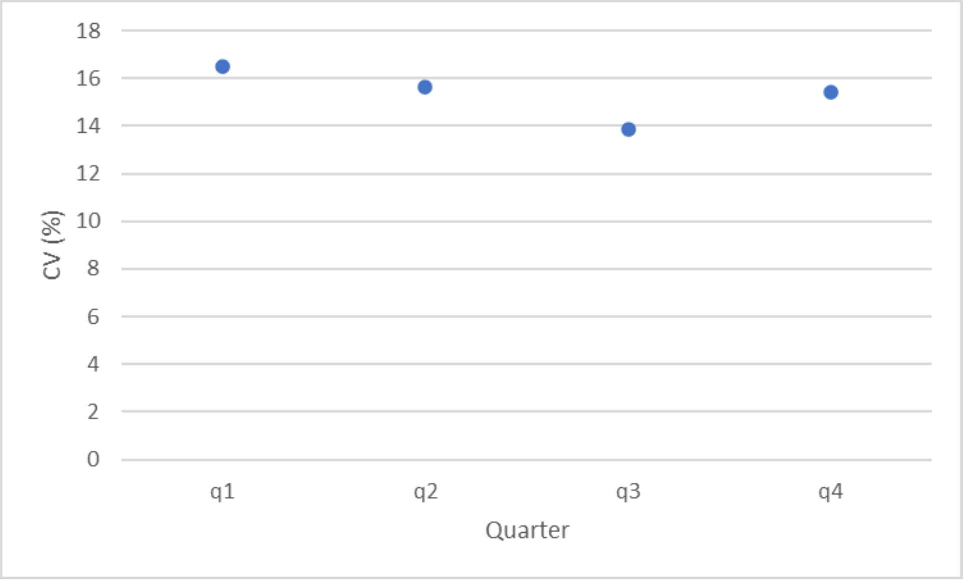

Figure S21 CVT by quarter for FVC

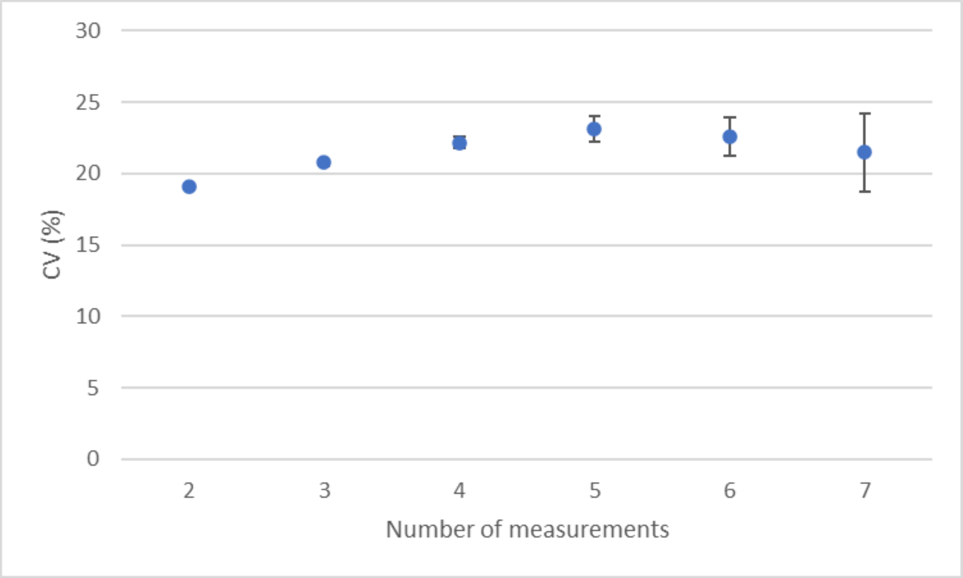

Figure S22 CVT by Number of measurements for FEV1

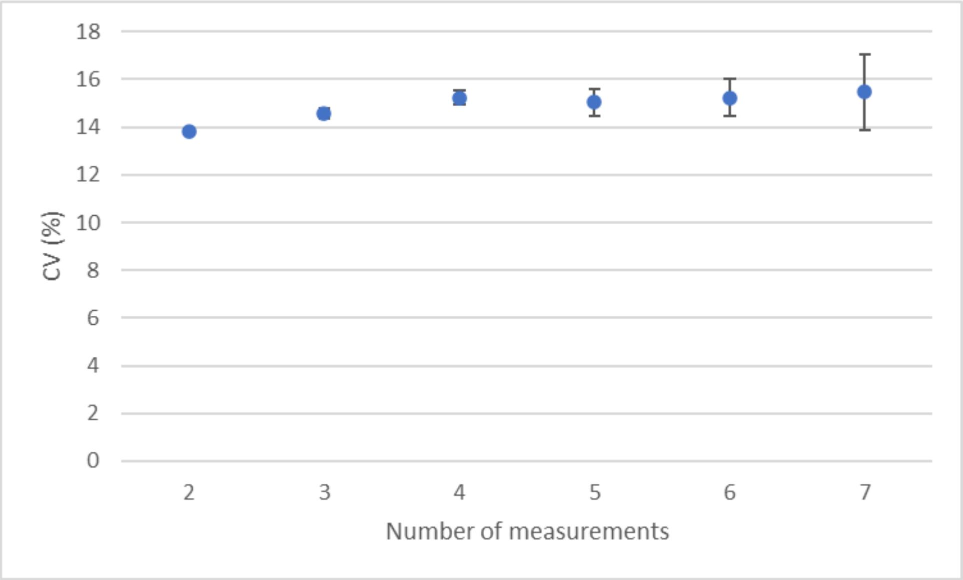

Figure S23 CVT by Number of measurements for FVC

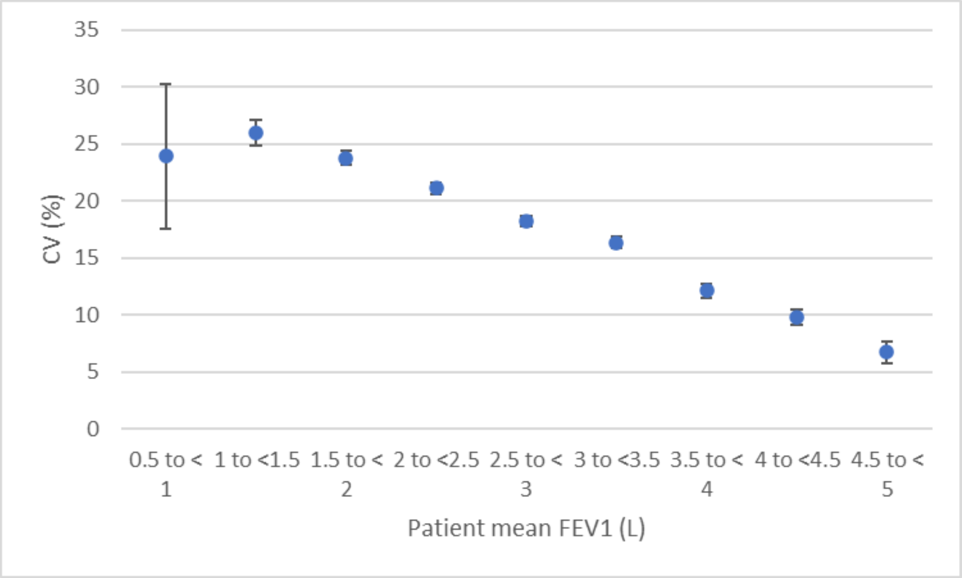

Figure S24 CVT by mean for FEV1

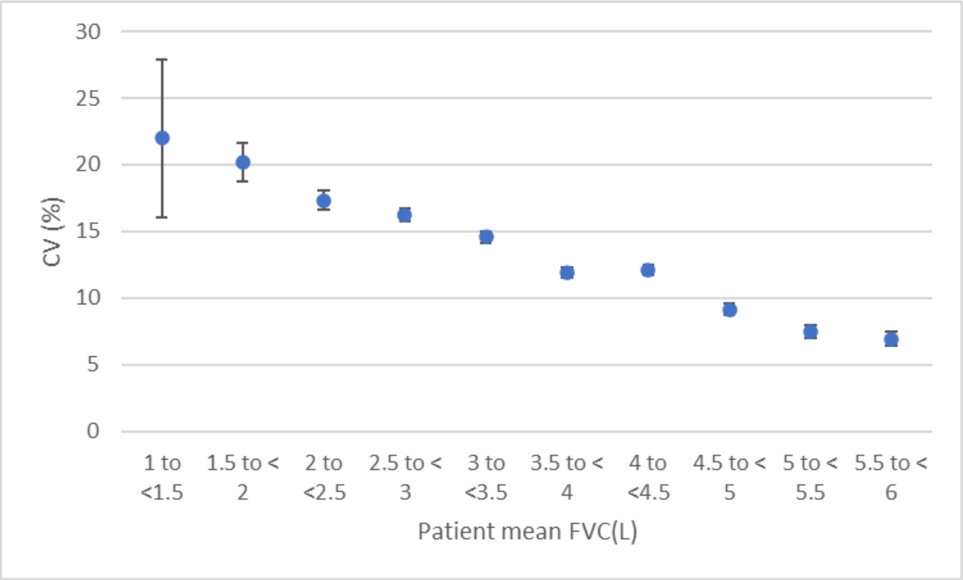

Figure S25 CVT by mean for FVC

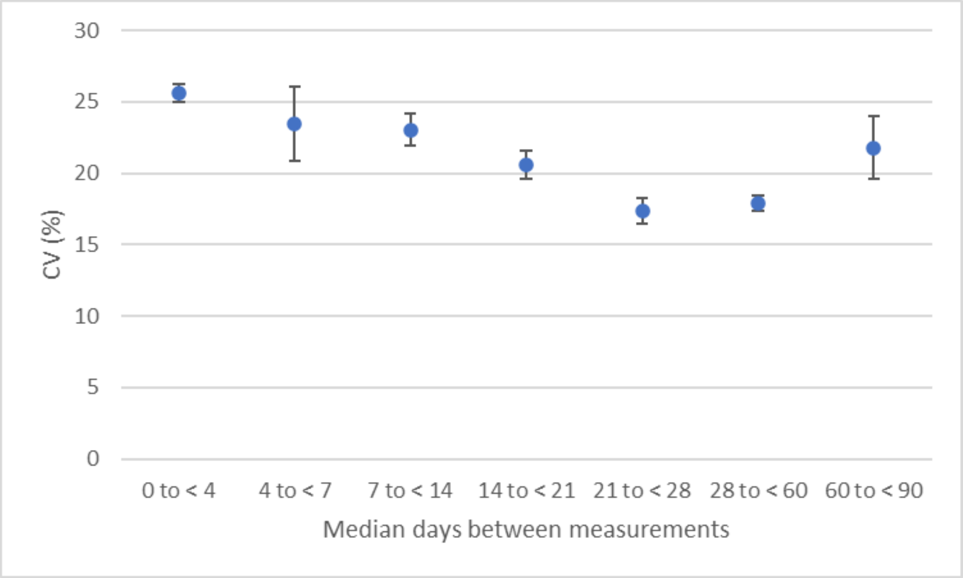

Figure S26 CVT by days between measurements for FEV1

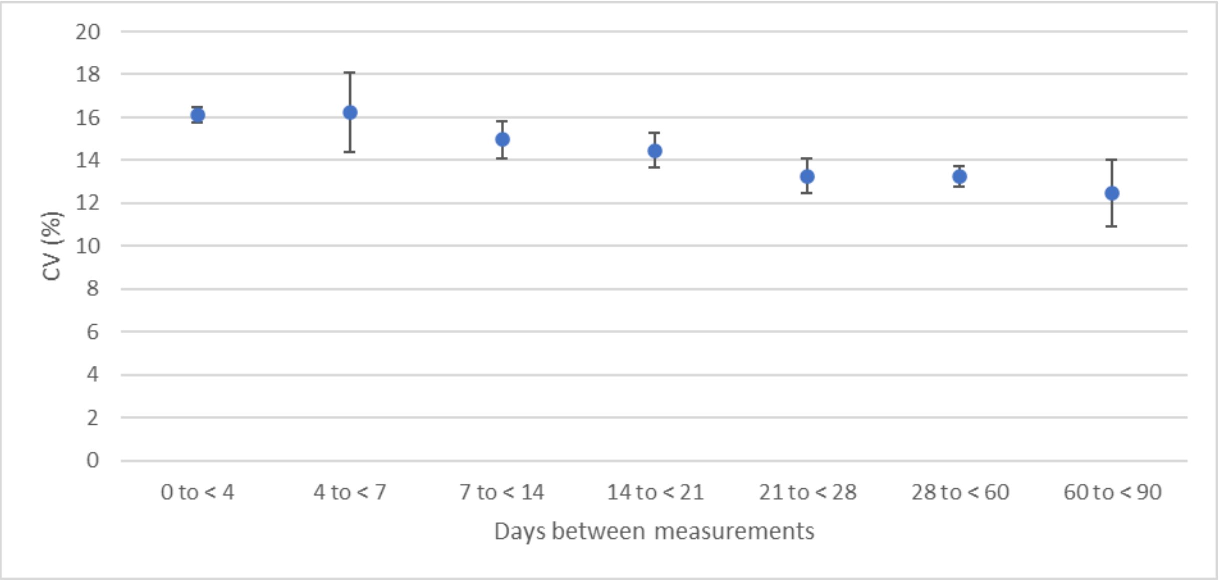

Figure S27 CVT by days between measurements for FVC

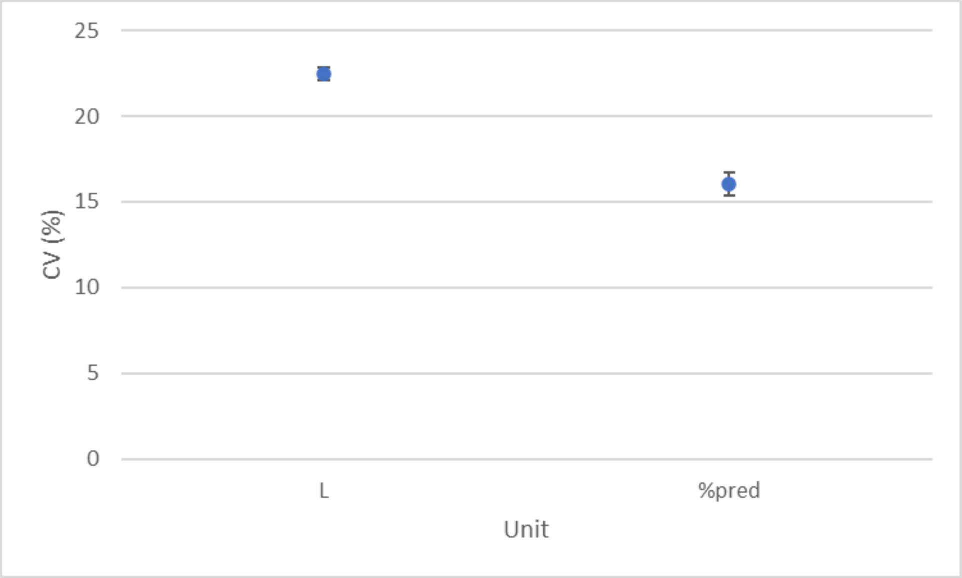

Figure S28 CVT by unit of measurement for FEV1

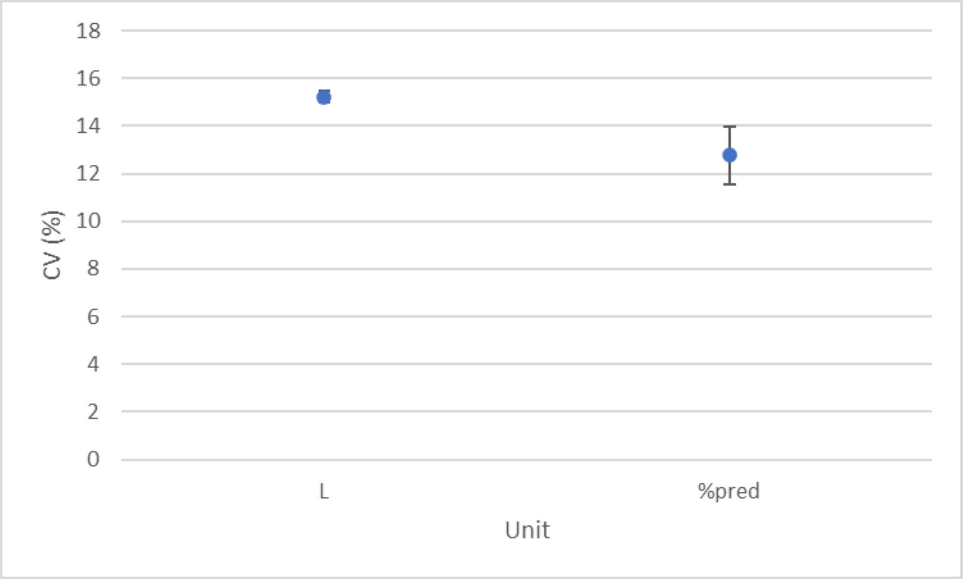

Figure S29 CVT by unit of measurement for FVC

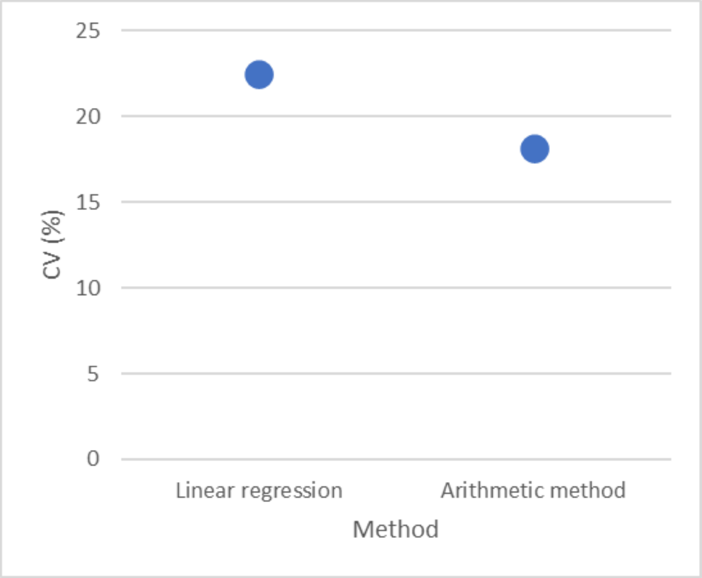

Figure S30 CVT by arithmetic calculation methods for FEV1

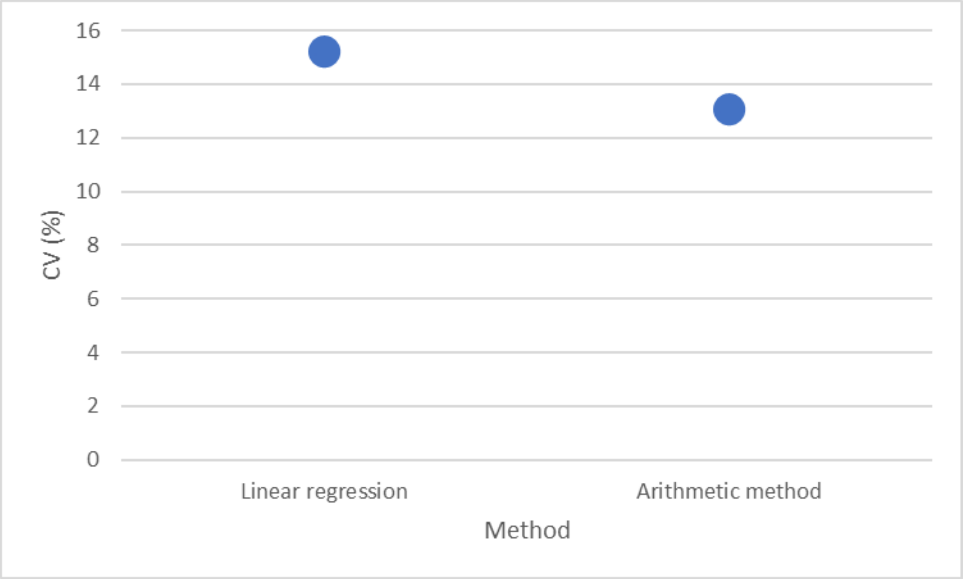

Figure S31 CVT by arithmetic calculation methods for FVC

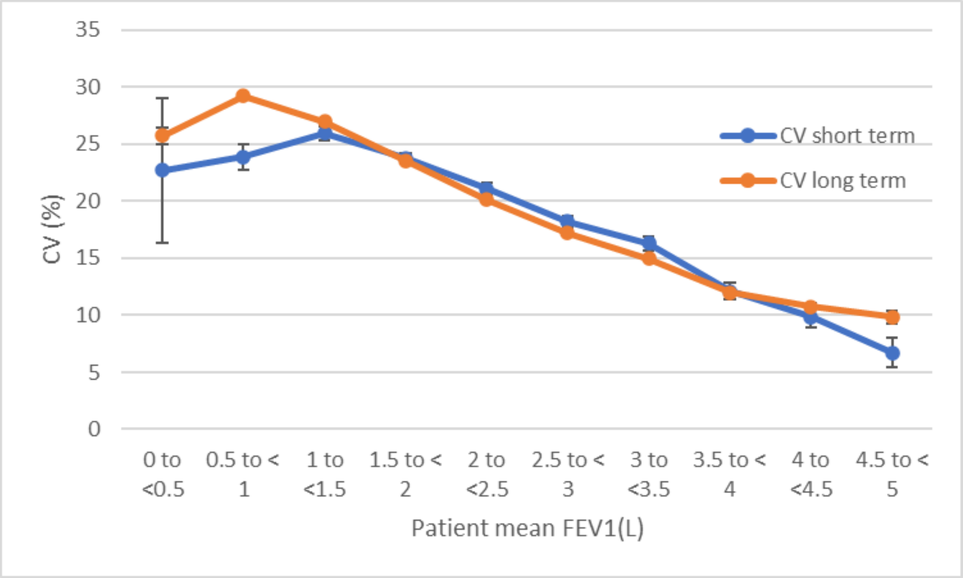

Figure S32 CVT by mean short and long-term data for FEV1

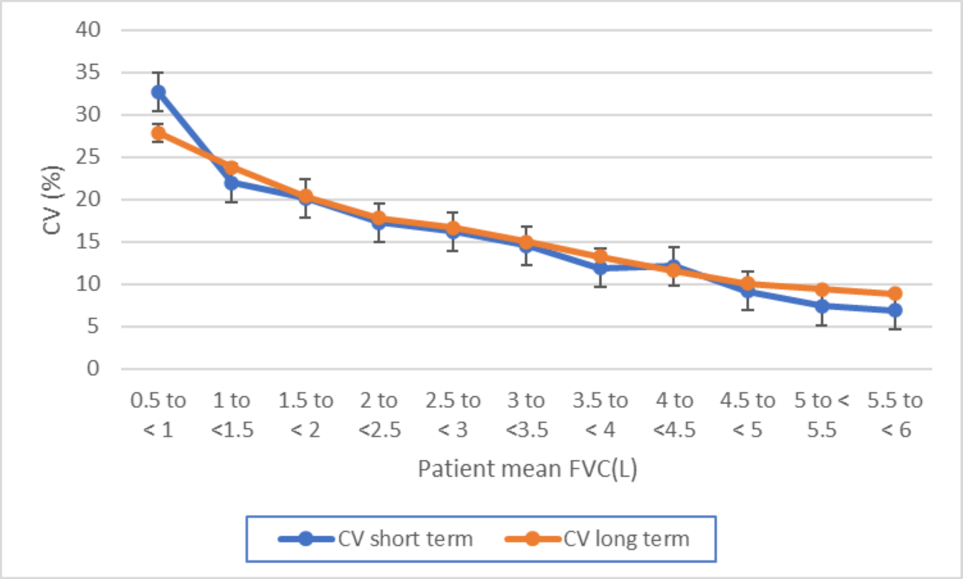

Figure S33 CVT by mean short and long-term data for FVC

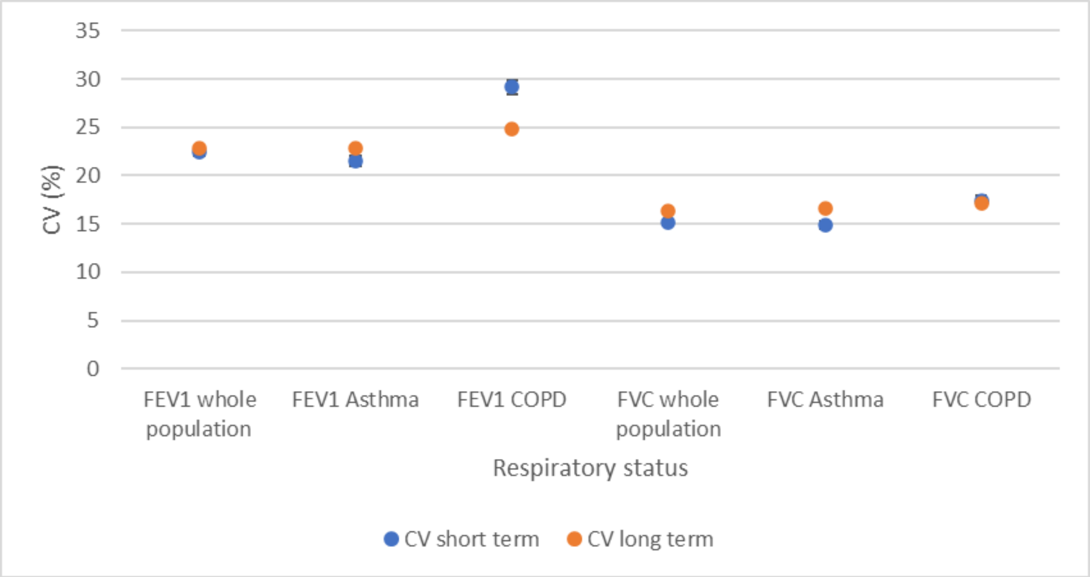

Figure S34 CVT by respiratory status for short and long-term data for FEV1 and FVC

Table S5 NICE guidelines for categorising severity of airflow obstruction.

FEV1 % predicted	NICE guideline CG12 (2004) severity of airflow obstruction
≥ 80%	Not categorised
50–79%	Mild
30–49%	Moderate
< 30%	Severe

Table S6 Variables extracted from database.

Variables extracted	
Test characteristics	
	Date of measurement
	Unit of measurement
Patient characteristics	
Sociodemographic	Age
	Sex
	Ethnicity
	Townsend Deprivation Score
	BMI
	Geographical region
Lifestyle factors	Smoker status
	Alcohol consumption status
Diagnoses and comorbidities	Prediabetes
	Type one diabetes mellitus
	Type two diabetes mellitus
	Diabetic medications
	Hypertension
	Hyperthyroidism
	Hypothyroidism
	Ischaemic heart disease
	Heart failure
	Ischaemic stroke
	Haemorrhagic stroke
	All cancers

Table S7 Sensitivity analysis for number of measurements in short and long term data, for FEV1

Measurement frequency	*CVT*
≥4 measurements in 6 months	*22.4*
2 measurements in 6 months	*19.1*
3 measurements in 6 months	*20.8*
≥4 measurements any time period	*22.8*
2 measurements any time period	*16.6*
3 measurements any time period	*18.6*

Table S8 Sensitivity analysis for number of measurements in short and long term data, for FVC

Measurement frequency	*CVT*
≥4 measurements in 6 months	*15.2*
2 measurements in 6 months	*13.8*
3 measurements in 6 months	*15.6*
≥4 measurements any time period	*16.4*
2 measurements any time period	*15.3*
3 measurements any time period	*15.5*

Table S9. Thresholds to identify whether the true change in the direction observed

		Is the true change in the direction I observed? (one-tail)		
	95% CI for a single measurement's deviation from the true value, repeatability coefficient (2.77 x SDw)	80% confidence level (4/5) z-score = 0.84	90% Confidence level (9/10) z-score = 1.28	95% Confidence (19/20) z-score = 1.64
**Those without Disease**				
FEV1	1.034	0.443	0.676	0.866
FVC	1.230	0.528	0.804	1.030
FEV1/FVC	0.175	0.075	0.115	0.147
**Asthma**				
FEV1	1.227	0.526	0.802	1.027
FVC	1.272	0.545	0.831	1.065
FEV1/FVC	0.338	0.145	0.221	0.283
**COPD**				
FEV1	1.389	0.596	0.908	1.163
FVC	1.355	0.581	0.886	1.135
FEV1/FVC	0.387	0.166	0.253	0.324
**Asthma and COPD overlap**				
FEV1	1.332	0.571	0.871	1.116
FVC	1.372	0.588	0.896	1.149
FEV1/FVC	0.298	0.128	0.195	0.250

Appendix 3

Sample statistical code for calculation of CV_T

```
drop if measure_unit != "MEA154"

drop if num_result == .

drop if num_result == 0

drop if num_result < 0

drop if num_result > 10


sort practice_patient_id

egen idnum = group ( practice_patient_id)


gen eventdatenum = date(event_date, "YMD")

bysort practice_patient_id: drop if eventdatenum == eventdatenum[_n-1]

. gen eventdate = date(event_date, "YMD")

. format eventdate %td

. sort idnum eventdate

. by idnum: gen eventdateinterval = eventdate - eventdate[_n-1]

. egen medianeventinterval = median(eventdateinterval), by(idnum)
```

```
gen birthdate1 = date( year_of_birth, "YMD")

format birthdate1 %td

personage birthdate1, currdate (mdy(5, 1, 2020)) generate (age)


sort idnum

by idnum: generate measnuma = _N

drop if measnuma < 4

egen sdnum_result = sd(num_result), by(idnum)

egen meannum_result = mean(num_result), by(idnum)

gen num_resultCV = sdnum_result/meannum_result

sum num_resultCV

drop if num_resultCV == 0

sort idnum

by idnum: generate measnum = _N

drop if measnum < 4

egen total_observations = count(num_result), by(idnum)

drop if total_observations < 4

replace bmi2 = . if bmi2 < 14

replace bmi2 = . if bmi2 > 70
```

Less than 6 months apart

```
sort idnum

. gen eventdate1 = date( event_date, "YMD")

by idnum: generate measrow = _n

by idnum: generate firsteventdate = eventdate1 if measrow == 1

by idnum: egen firstevendate2 = min(firsteventdate)

generate new_date = firstevendate2 + 180

format new_date %td

format firstevendate2 %td

drop if eventdate > new_date


sort idnum

by idnum: generate measnumb = _N

drop if measnumb < 4
```

\\\Whole population

```
mixed num_result || idnum: , reml

matrix list e(V)
matrix list e(b)
nlcom (est_mean: [num_result]_cons )
nlcom (est_varg: (exp([lns1_1_1]_cons))^2)
nlcom (est_vari: (exp([lnsig_e]_cons))^2)
nlcom (est_sdg: (exp([lns1_1_1]_cons)))
nlcom (est_sdi: (exp([lnsig_e]_cons)))
nlcom (est_cvi: ((exp([lnsig_e]_cons)))/([num_result]_cons))
```

By sex

```
mixed num_result if sex == "F" || practice_patient_id : , reml
matrix list e(V)
matrix list e(b)
nlcom (est_mean: [num_result]_cons )
```

```
nlcom (est_varg: (exp([lns1_1_1]_cons))^2)

nlcom (est_vari: (exp([lnsig_e]_cons))^2)

nlcom (est_sdg: (exp([lns1_1_1]_cons)))

nlcom (est_sdi: (exp([lnsig_e]_cons)))

nlcom (est_cvi: ((exp([lnsig_e]_cons)))/([num_result]_cons))


mixed num_result if sex == "M" || practice_patient_id : , reml

matrix list e(V)

matrix list e(b)

nlcom (est_mean: [num_result]_cons )

nlcom (est_varg: (exp([lns1_1_1]_cons))^2)

nlcom (est_vari: (exp([lnsig_e]_cons))^2)

nlcom (est_sdg: (exp([lns1_1_1]_cons)))

nlcom (est_sdi: (exp([lnsig_e]_cons)))

nlcom (est_cvi: ((exp([lnsig_e]_cons)))/([num_result]_cons))
```
